# An Evidence Synthesis of Covert Online Strategies Regarding Intimate Partner Violence

**DOI:** 10.1177/1524838020957985

**Published:** 2020-09-15

**Authors:** Aikaterini Grimani, Anna Gavine, Wendy Moncur

**Affiliations:** 1 Warwick Business School, 3042 University of Warwick, Coventry, United Kingdom; 2 School of Nursing and Health Sciences, 3042 University of Dundee, United Kingdom; 3 Computer & Information Sciences, 3042 University of Strathclyde, Glasgow, United Kingdom

**Keywords:** intimate partner violence, covert behaviors, social networking sites, digital technologies, interpersonal electronic surveillance

## Abstract

This systematic review synthesizes evidence of how people use the internet to deploy covert strategies around escaping from, or perpetrating, intimate partner violence (IPV). Online tools and services can facilitate individuals leaving abusive relationships, yet they can also act as a barrier to departure. They may also enable abusive behaviors. A comprehensive literature search of published and unpublished studies in electronic databases was conducted. Two researchers independently screened abstracts and full texts for study eligibility and evaluated the quality of included studies. The systematic review includes 22 studies (9 qualitative and 11 cross-sectional studies, a randomized control trial [RCT] and a nonrandomized study [NRS]) published between 2004 and 2017. Four covert behaviors linked to covert online strategies around IPV were identified: presence online, granular control, use of digital support tools and services, and stalking and surveillance. The same technology that provides individuals with easy access to information and supportive services related to IPV, such as digital devices, tools, and services, also enables perpetrators to monitor or harass their partners. This review takes a rigorous interdisciplinary approach to synthesizing knowledge on the covert strategies adopted by people in relation to IPV. It has particular relevance to practitioners who support survivors in increasing awareness of the role of digital technologies in IPV, to law enforcement agencies in identifying new forms of evidence of abuse, and in enabling designers of online/social media applications to take the needs and vulnerabilities of IPV survivors into account.

This article reports on an interdisciplinary systematic review conducted to identify the covert strategies deployed online by individuals involved in intimate partner violence (IPV), either perpetrators or survivors, and the enablers and barriers encountered in using these strategies. The research was carried out as part of the Keeping Secrets Online project (crestresearch.ac.uk/projects/keeping-secrets-online/), which synthesizes new knowledge of how people use the internet to facilitate secret-keeping in a range of contexts.

The topic of experiencing or perpetrating IPV was selected as a rich area of study as there is a high level of motivation for people to keep secrets online in this context. IPV is defined as any behavior within an intimate relationship that causes physical, psychological, or sexual harm to those in the relationship (Krug et al., 2002). A victim^1^ may seek support online in coping with IPV or assistance and advice in escaping from it. If the perpetrator discovers their activities, the victim may be at risk of physical and psychological harm from the perpetrator, or the victim may be deterred from escaping from the abusive relationship. Technology-based IPV creates emotional turmoil, life complications, and helplessness, especially in women’s lives (Brown et al., 2018).

## Victims/Survivors of IPV

Women aged 18–29 are at higher risk of IPV than older women (Bradley & Potter, 2018; Brown et al., 2018). Although some studies describe equal rates of IPV perpetration across genders or imply that women more often perpetrate some forms of IPV, researchers adopting feminist epistemological approaches emphasize the importance of looking at the context and consequences around IPV before concluding that there is gender symmetry (Brown et al., 2018).

There have been attempts made to educate those either experiencing or at risk of IPV about internet safety (Finn & Atkinson, 2009) and to deliver online interventions that aim to reduce their risk. Online interventions include the use of internet-based safety decision aids and action plans, which can be accessed through smartphones, tablets, and computers (Bloom et al., 2014; Eden et al., 2015; Ford-Gilboe et al., 2017; Koziol-McLain et al., 2015; Tarzia et al., 2016; Wathen & McKeown, 2010). Victims and survivors can access online tools that help increase privacy and safety, while online fora can support their emotional and psychological recovery (Brem et al., 2017; Jenaroa et al., 2018; Lee & Anderson, 2016; Marganski & Melander, 2018; Melander, 2010; Southworth et al., 2007).

In this systematic review, we sought to identify and synthesize the strategies used by individuals who had experienced any form of IPV (including physical violence, coercive control, cyberstalking) and had engaged in covert online behaviors when attempting to leave a relationship. This included generating a new identity or concealing an old identity online. We also considered how online tools and services serve as a means of extending abusive behaviors by the perpetrator.

## Perpetrators

Perpetrators’ efforts to exert power and control by frightening, stalking, monitoring, and controlling their victims have been enabled by a wide range of technological tools. These tools range from early innovations such as caller identification, fax machines, calling cards, and cordless telephones to more contemporary ones such as cellular and wireless telephones, GPS and location services, spyware software and keystroke login hardware, and hidden cameras (Al-Alosi, 2017; Melander, 2010; Southworth et al., 2007). Social networking sites afford unique opportunities to perpetrators to humiliate, manipulate, or harass their victim, within an online community that is typically occupied by the victim’s friends and family (Brown et al., 2018; Moncur & Herron, 2018).

*Surveillance* and “monitoring” are terms that are used interchangeably by researchers. Monitoring is often reported as the most common form of technology-based IPV used by perpetrators and has been defined as “the use of ICTs to gather information about a romantic partner that creates or enhances a dynamic of control within the relationship” (Brown et al., 2018, p. 215). Interpersonal electronic surveillance is characterized as “surreptitious strategies individuals use over communication technologies to gain awareness of another user’s offline and/or online behaviours” (Tokunaga, 2011, p. 706). Surveillance and monitoring may be carried out by partners involved in intimate relationships (even those not involving IPV) as a strategy in response to threats of extradyadic rivals, or in the early or intermediate stages of a new relationship, to obtain more information about the other (Tokunaga, 2011).

Technology-enabled abusive behaviors enacted by perpetrators may include cyberstalking—unwelcome and intrusive behaviors that involve repeated threats and/or harassment via email or other computer-mediated communication (Henry & Powell, 2018; Powell & Henry, 2016; Smoker & March, 2017; Southworth et al., 2007); *fraping*—“an activity that involves the unauthorised alteration of information on an individual’s online social network site profile by a third party” (Moncur et al., 2016, p. 125); monitoring email communication either directly on the victim’s computer or through “sniffer” programs (pieces of software that collect access codes that allow entry into a targeted system); sending insulting emails; disrupting email communications by flooding a victim’s email inbox with unwanted mail; or by sending a virus program (Marganski & Melander, 2018; Melander, 2010; Moncur et al., 2016; Southworth et al., 2007).

These abusive behaviors are an extension of common—albeit undesirable—online behaviors enacted in romantic relationships. In one survey, over 65% of adults used technology to monitor a partner (e.g., hacking into a partner’s email; Burke et al., 2011). In a later survey, 43% of men monitored their partner’s social interactions through common technological sources (e.g., mobile phone, email, and social networks) by gaining access to their password-protected information, while over 15% of men used GPS technology to monitor a partner’s activities (Brem et al., 2017). Moreover, Leisring and Giumetti (2014) found that 93% of college students both perpetrated and experienced minor cyber abuse (e.g., swearing at or insulting partner) involving their partner, while 13% perpetrated and experienced severe cyber abuse (e.g., threats, public humiliation).

## Objectives

The purpose of this systematic review was to identify how individuals either experiencing or perpetrating IPV engage in covert online behaviors. Specifically, the following research questions were addressed.

**Research Question 1:** What covert online strategies do survivors use in relation to IPV?
**Research Question 2:** What strategies do perpetrators use online to covertly extend their abusive behaviors?
**Research Question 3:** How are the strategies identified in Research Question 1 and Research Question 2:

- affected by age?
- affected by gender?
- varied across non-Western and diaspora populations?

## Method

A systematic review was conducted, as this affords a more robust approach to search, appraisal, and synthesis of the literature than traditional reviews. Systematic reviews were originally developed for use in medical research; however, they are now used in a range of different disciplines (Haddaway & Bilotta, 2016). The protocol for this systematic review was registered in the International Prospective Register of Systematic Reviews (with Registration Number CRD42018091691).

### Inclusion/Exclusion Criteria

Studies were included in the review if they met the following criteria:

- Quantitative or qualitative research studies that present empirical methods and results;
- Explored internet use, either by individuals who have experienced IPV, in order to facilitate protection from perpetrators and support from friends, family, and professionals *or* by perpetrators as a means of control, surveillance, and harassment;
- Included adults aged over 16 who have experienced violence (physical, sexual, emotional) from their intimate partner, or perpetrated IPV (no restrictions were placed on gender, geographical region, or sexuality);
- Written in English language;
- Published from 2004 to current (searches conducted February 2018). We considered literature linked to early, as well as current, use of social networking sites (SNSs). While Facebook was released in 2004, MySpace was the largest SNS in the world from 2005 to 2008, while others were also popular—for example, Friendster, Bebo, and Cyworld. Facebook became the most popular SNS globally in 2009.

Studies were excluded in the review if they:

- Did not report empirical methods and results (e.g., commentaries, editorials),
- Included children and young people under 16 years,^2^
- Included adults who experienced sexual violence or harassment from somebody that was not an intimate partner,
- Did not explore the use of the internet in the context of IPV,
- Were not published in English,
- Were published before 2004: Social media, and in particular SNSs such as Facebook, became ubiquitous and started radically altering the nature and scope of social interaction for their users (e.g., self-presentation, publicly disclosed information, surveillance by audiences) after 2004.

### Search Strategy and Selection Process

A series of steps were undertaken in identifying relevant papers. These comprised creating and running a search strategy, screening abstracts and titles, evaluating methodological quality of each study, extracting relevant data from each study screened successfully, and developing a narrative synthesis of the findings from the included studies. Each step is described in turn below.

A *search strategy*, using Medical Subject Headings (MeSH) terms and relevant key words, was developed (Supplemental Material Table B). The search strategies included combining terms related to IPV with terms related to internet use with Boolean operators. No restrictions were placed on the search in terms of place of publication.

The following databases were searched: MEDLINE (via Ovid), Social Science Citation Index (via Web of Science), ASSIA (via ProQuest), PsycINFO (EBSCO), and ACM Digital Library. In addition, Google Scholar was searched, with results capped at the first 100 records, sorted by relevance. Gray literature was sought by manually searching the following websites relevant to the topic area: World Health Organization, United Nations Women, End Violence Against Women, Department for International Development, PEW Research Centre. Editorials, letters, working papers, reports, and reviews were excluded. Finally, in order to ensure no relevant studies were omitted, additional studies were identified from the reference lists of studies that met the inclusion criteria and were included in the review.

All studies identified by the search were imported into Endnote 7, and duplicates were removed. Two reviewers (A.Gr. and A.Ga.) independently screened all titles and abstracts against the eligibility criteria. At this stage, we were purposefully overinclusive and only excluded any obviously irrelevant studies. The full texts of studies potentially meeting the eligibility criteria were then retrieved and screened independently by A.Gr and A.Ga. against the eligibility criteria. Differences in judgment at both stages were resolved through a consensus procedure. A record was kept of all discarded full-text articles, including the reason for discard.

The two reviewers independently evaluated *the methodological quality* of each study, using an assessment tool appropriate to the study design. Discrepancies were resolved through a consensus procedure. Due to the methodological diversity of the included research studies, a range of appraisal tools were necessary to assess different study designs and included:

- Critical Appraisal Skills Program Checklist for qualitative studies (Dixon-Woods et al., 2007; Walsh & Downe, 2006),
- Appraisal tool for cross-sectional studies (Downes et al., 2016),
- Cochrane Collaboration Risk of Bias Tool for Randomized controlled trials (Armijo-Olivo et al., 2012; Higgins et al., 2011),
- Quality Assessment Tool for Quantitative Studies (Armijo-Olivo et al., 2012; National and Collaborating Centre for Methods, 2008).

A *data extraction* form was developed, reviewed, and refined by the researchers and includes the following: information on publication (title, authors, year), study aims, geographical location, context and setting, sampling approach, ethical issues, participant characteristics, data collection methods), data analysis approach, data collected, and results. One reviewer extracted the data (A.Gr.), while a second reviewer (A.Ga.) checked all the extracted data.

A *narrative synthesis* of the findings from the included studies, and the structures around the type of studies (experimental, survey, ethnography, etc.), was conducted. This approach is flexible, allowing for different types of evidence, both qualitative and quantitative, to be synthesized (Mays et al., 2005; Popay et al., 2006). The following stages of analysis were used to develop the synthesis. First, *content analysis* was used to identify different clusters/groupings of covert strategies, the frequency with which these strategies are employed and the extent to which they are effective in maintaining privacy. Content analysis is a systematic, replicable technique for compressing many words of text into fewer content categories based on explicit rules of coding (Stemler, 2001). It is also useful for examining trends and patterns in documents (Mays et al., 2005; Popay et al., 2006). The process of creating codes was a combination of both predetermined (a priori) and emergent coding. Predetermined coding was based on a previous coding dictionary from other relevant research studies and key concepts, while emergent coding was based on concepts, actions, or meanings that evolved from the data and were different from the predetermined codes (Stemler, 2001).

Secondly, *thematic analysis* of the data, the most common method adopted within narrative reviews, was used to systematically identify the main, recurrent, or most important themes or concepts across the included studies. The following three stages were conducted: coding text, developing descriptive themes, generating analytical themes (Thomas & Harden, 2008). As a method, it provides a means of organizing and summarizing the findings from large, diverse bodies of research (Mays et al., 2005; Popay et al., 2006). NVivo (Version 12.0) qualitative software was used to facilitate analysis. It provides a robust and pragmatic way to manage the complexities of conducting qualitative evidence synthesis, facilitates framework synthesis, and provides clear an audit trail, enhancing confidence in synthesis findings (Houghton et al., 2017).

Thirdly, the findings of these analyses for each study were then compared using a process known as translation (France et al., 2019). Translation enables common themes from across the studies to be identified and then synthesized narratively. The synthesis goes beyond simple reporting of individual study findings and aims to bring together the combined findings of all the studies using a textual approach. Finally, the robustness of the narrative synthesis was assessed by considering the quality of the evidence related to the research findings and for drawing conclusions about the strategies (Popay et al., 2006).

## Results

**Figure 1.**
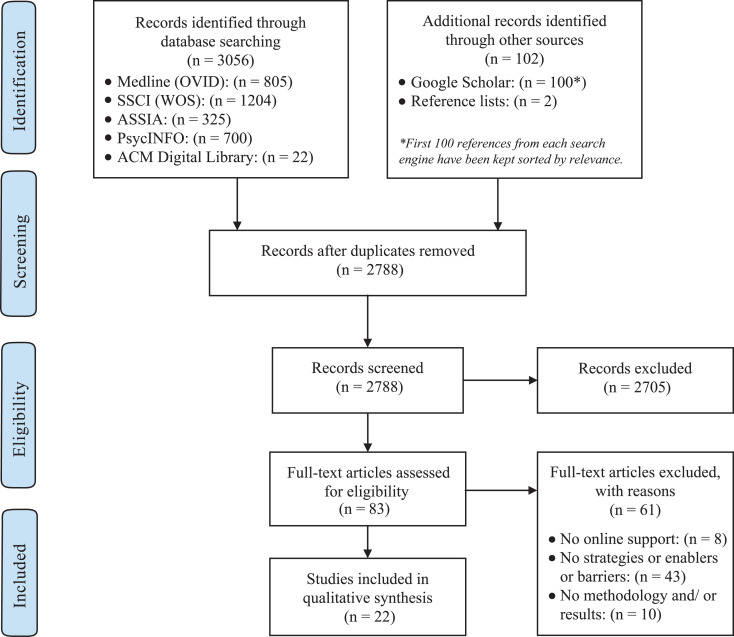
Flowchart of the study selection process used for the systematic review.

Using the search strategy and selection process described above resulted in only 22 articles being retained from an initial set of 3,158 citations (see Figure 1), with the result set incrementally reduced as follows: (i) The search of the predefined databases resulted in 3,056 records. (ii) A further 102 records were found in other sources, giving a total of 3,158 citations. The latter included references from relevant studies, reviews, and publications from Google Scholar. (iii) After duplicates were removed (*n* = 370), a total of 2,788 citations were screened against the inclusion criteria. (iv) Of these, 2,705 citations were excluded on the basis of title, key words, and abstract. (v) The full texts of the remaining articles (*n* = 83) were then assessed against the inclusion criteria, resulting in 22 articles being retained. The reasons for exclusion are presented in Figure 1. Of the 22 studies retained (Supplemental Material Table A1), nine were qualitative, 11 were cross-sectional studies, one study was an RCT, and one study was an NRS. The majority of studies were conducted in the United States (*n* = 19), while one was conducted in Canada and two in Australia. Sample size ranged from 6 to 1,683 participants (6,932 in total; mean sample size: 315.1; median sample size: 112).

### Strategies Used

The narrative synthesis described above served to identify three strategies that satisfied Research Question 1—that is, they were used as covert strategies used by survivors in relation to IPV: *presence online, granular control,* and *use of digital support tools and services*. One strategy was identified that satisfied Research Question 2: *stalking and surveillance*, which was used by perpetrators. All strategies are detailed in Table 1 (see also Supplemental Material Table A2) and expanded upon below.

**Table 1.** Covert Strategies Related to IPV.

Covert Strategies	No. of Studies and References	
Presence online	9	(Bosch & Schumm, 2004; Chaulk & Jones, 2011; Choo et al., 2015; Dimond et al., 2011; Freed et al., 2017; Halligan et al., 2013; Lindsay et al., 2013; Matthews et al., 2017; Tarzia et al., 2017)
Granular control	7	(Chaulk & Jones, 2011; Dimond et al., 2011; Finn & Atkinson, 2009; Freed et al., 2017; Matthews et al., 2017; Tarzia et al., 2017; Truman, 2011)
Technological interventions	8	(Bacchus et al., 2016; Bloom et al., 2014; Choo et al., 2015; Constantino et al., 2007; Finn & Atkinson, 2009; Freed et al., 2017; Lindsay et al., 2013; Tarzia et al., 2017)
Stalking and surveillance	14	(Brem et al., 2015; Burke et al., 2011; Chaulk & Jones, 2011; Dimond et al., 2011; Finn & Atkinson, 2009; Fox & Tokunaga, 2015; Freed et al., 2017; Marcum et al., 2017; Matthews et al., 2017; Reed et al., 2016; Ross et al., 2016; Rothman et al., 2009; Truman, 2011; Woodlock, 2017)

*Note*. IPV = intimate partner violence.

Digital devices (such as smartphones, computers, tablets, GPS devices, digital cameras), tools, and services (such as web/mobile applications, software, blocking/monitoring tools, spyware) enabled these strategies, by providing those experiencing IPV with easy access to information, and opportunities for professional, peer support, and concealment from perpetrators. However, these same devices, tools, and services were also used by perpetrators in monitoring or harassing their partners and in deterring supportive behaviors (see Tables 2 and 3; Supplemental Material Tables A3 and A4). Thus, digital technologies can be helpful, but SNSs such as Facebook do not easily provide the kind of privacy that victims of IPV require. A summary table of critical findings is also provided below (see Table 4).

**Table 2.** Technology Which Enables Concealed and Supportive Behaviors Related to IPV.

Facilitators	No. of Studies and References	
Digital devices	8	(Bacchus et al., 2016; Bloom et al., 2014; Bosch & Schumm, 2004; Choo et al., 2015; Constantino et al., 2007; Finn & Atkinson, 2009; Lindsay et al., 2013; Tarzia et al., 2017)
Digital tools and services	6	(Finn & Atkinson, 2009; Freed et al., 2017; Lindsay et al., 2013; Matthews et al., 2017; Tarzia et al., 2017; Truman, 2011)

*Note*. IPV = intimate partner violence.

**Table 3.** Technology Which Deters Concealed and Supported Behaviors Related to IPV.

Barriers	No. of Studies and References	
Social networking sites	8	(Brem et al., 2015, Burke et al., 2011, Chaulk & Jones, 2011, Dimond et al., 2011, Fox & Tokunaga, 2015, Freed et al., 2017, Marcum et al., 2017, Woodlock, 2017)
Digital devices	9	(Bacchus et al., 2016, Burke et al., 2011, Choo et al., 2015, Dimond et al., 2011, Freed et al., 2017, Halligan et al., 2013, Reed et al., 2016, Truman, 2011, Woodlock, 2017)
Digital tools and services	7	(Burke et al., 2011; Dimond et al., 2011; Freed et al., 2017; Matthews et al., 2017; Rothman et al., 2009; Truman, 2011; Woodlock, 2017)

*Note*. IPV = intimate partner violence.

**Table 4.** Summary of Critical Findings.

Strategies	Facilitators to Effective Implementation of the Strategies	Barriers to Effective Implementation of the Strategies
Presence online Restricted present online/use of technology (Dimond et al., 2011; Freed et al., 2017; Matthews et al., 2017) Use of communication channels (Chaulk & Jones, 2011; Dimond et al., 2011; Halligan et al., 2013) Access to supportive resources (Bosch & Schumm, 2004; Choo et al., 2015; Lindsay et al., 2013; Tarzia et al., 2017)	Digital devices Smartphone (Lindsay et al., 2013; Tarzia et al., 2017) Computer with access to the internet/email (Bosch & Schumm, 2004)	Social networking sites Facebook (Chaulk & Jones, 2011; Dimond et al., 2011; Freed et al., 2017)
	Digital tools and services Web applications (Tarzia et al., 2017) Mobile application (Lindsay et al., 2013) Software (Matthews et al., 2017)	Digital devices GPS device; computer; mobile phone (Dimond et al., 2011; Freed et al., 2017; Halligan et al., 2013)
		Digital tools and services Blocking tools (Dimond et al., 2011) Monitoring tools (Freed et al., 2017) Spyware (Matthews et al., 2017)
Granular control Anonymous email accounts (Dimond et al., 2011; Finn & Atkinson, 2009) Blocking mechanisms (Dimond et al., 2011; Freed et al., 2017; Matthews et al., 2017; Truman, 2011) Strengthened privacy settings (Chaulk & Jones, 2011; Finn & Atkinson, 2009; Freed et al., 2017; Matthews et al., 2017; Tarzia et al., 2017) Limited or avoiding sharing information online (Dimond et al., 2011; Freed et al., 2017; Matthews et al., 2017)	Digital devices Computer (Finn & Atkinson, 2009) Smartphone (Tarzia et al., 2017)	Social networking sites Facebook and other social media (Chaulk & Jones, 2011; Dimond et al., 2011; Freed et al., 2017)
	Digital tools and services Phone and computer-related services (Finn & Atkinson, 2009) Google search (Freed et al., 2017) Caller ID or call blocking (Truman, 2011) Software (Matthews et al., 2017) Web applications (Tarzia et al., 2017)	Digital devices GPS device; computer; mobile phone (Dimond et al., 2011; Freed et al., 2017) Video or digital cameras; GPS device; listening devices (Truman, 2011) Mobile phone (Freed et al., 2017)
		Digital tools and services Blocking tools (Dimond et al., 2011) Spyware (Matthews et al., 2017; Truman, 2011) Monitoring tools (Freed et al., 2017)
Use of digital support tools and services Digital interventions: Online Safety Planning Intervention (Bloom et al., 2014); Technology Safety Project (Finn & Atkinson, 2009); DOVE technology (Bacchus et al., 2016); Computer Interventions (Choo et al., 2015); e-mail device “MIVO” intervention (Constantino et al., 2007); Personalized safety plan (Lindsay et al., 2013) Online support services (Bloom et al., 2014; Finn & Atkinson, 2009; Freed et al., 2017; Lindsay et al., 2013; Tarzia et al., 2017)	Digital devices Computer; mobile phone; smartphone (Bloom et al., 2014; Choo et al., 2015; Finn & Atkinson, 2009; Lindsay et al., 2013; Tarzia et al., 2017) Tablet (Bacchus et al., 2016) MIVO (e-mail device) (Constantino et al., 2007)	Digital devices Computer (Choo et al., 2015; Freed et al., 2017) Tablet (Bacchus et al., 2016)
	Digital tools and services Phone and computer-related services/applications (Finn & Atkinson, 2009; Lindsay et al., 2013; Tarzia et al., 2017) Google search (Freed et al., 2017)	
Stalking and Surveillance Stalking (Brem et al., 2015; Burke et al., 2011; Chaulk & Jones, 2011; Dimond et al., 2011; Finn & Atkinson, 2009; Fox and Tokunaga, 2015; Freed et al., 2017; Matthews et al., 2017; Reed et al., 2016; Truman, 2011; Woodlock, 2017) Surveillance and monitoring (Burke et al., 2011; Dimond et al., 2011; Finn & Atkinson, 2009; Freed et al., 2017; Marcum et al., 2017; Matthews et al., 2017; Reed et al., 2016; Rothman et al., 2009; Truman, 2011; Woodlock, 2017) Sexting coercion (Ross et al., 2016)	Digital devices Computer (Finn & Atkinson, 2009)	Social networking sites Facebook and other social media (Brem et al., 2015; Burke et al., 2011; Chaulk & Jones, 2011; Dimond et al., 2011; Fox & Tokunaga, 2015; Freed et al., 2017; Marcum et al., 2017; Woodlock, 2017)
	Digital tools and services Phone and computer-related services (Finn & Atkinson, 2009) Google search (Freed et al., 2017) Software (Matthews et al., 2017) Caller ID or call blocking (Truman, 2011)	Digital devices GPS device; computer; mobile phone (Dimond et al., 2011; Freed et al., 2017) Monitoring devices (Burke et al., 2011; Reed et al., 2016; Woodlock, 2017) Video or digital cameras; GPS device; listening devices (Truman, 2011)
		Digital tools and services Monitoring tools (Burke et al., 2011; Freed et al., 2017) Email monitoring (Rothman et al., 2009) Spyware (Matthews et al., 2017; Truman, 2011; Woodlock, 2017)

### Presence Online

*Access to a computer* appears to have a protective effect, reducing abuse by giving the person experiencing IPV the opportunity to seek out supportive people who also offer information and advice (Bosch & Schumm, 2004). For some, a mobile device was their only connection to the outside world (Choo et al., 2015; Lindsay et al., 2013; Tarzia et al., 2017).

Online SNSs, such as Facebook, facilitate communication between friends and acquaintances and mediate the provision of information about activities, interests, and opinions among friends and acquaintances (Chaulk & Jones, 2011; Halligan et al., 2013). In times of isolation and separation from their social network, social media sites such as Facebook provide survivors with much needed *connection* to family and friends, and associated *social support*, even though survivors may have concerns about privacy (Dimond et al., 2011).

Conversely, three studies reported that survivors *restricted their presence online*, and access/use of technology (Dimond et al., 2011; Freed et al., 2017; Matthews et al., 2017). Some survivors avoided going online, for example, using a paper calendar, fearing that their abuser had greater technical prowess and could uncover their activities (Freed et al., 2017). Constraints were also placed on survivors’ online activities through perpetrators’ physical control of devices and monitoring behaviors (Dimond et al., 2011; Matthews et al., 2017).

### Granular Control

Survivors adopted more fine-grained strategies of control over their online presence, by concealing their identities and location, blocking contact from their perpetrators, strengthening privacy settings, restricting the content that they posted, and changing their digital devices in various ways.

Creation of *anonymous email accounts* protected survivors’ identities (Finn & Atkinson, 2009). Dimond et al. (2011) identified that survivors would register a new prepaid mobile phone under an alias to conceal their identity and could feel unable to use their real names again, as they feared that their information could show up on other phones or on the internet.

Survivors could also be proactive in using *blocking mechanisms:* for example, installing caller ID or call blocking to prevent the perpetrator contacting them, changing or installing new locks or security systems (Truman, 2011), or installing software that warns when someone is trying to hack into their accounts (Dimond et al., 2011; Freed et al., 2017; Matthews et al., 2017; Truman, 2011). However, attempts to evade contact with their perpetrator were made more challenging by difficulties in blocking unwanted calls and text messages, including financial costs and service provision by network carrier (Dimond et al., 2011).

Five studies reported the use of *strengthened privacy settings* as a way of achieving granular control over survivors’ online presence: for example, using Facebook privacy settings to restrict the majority of their profile to friends only, as well as to block some individuals and to limit profile viewing to others (Chaulk & Jones, 2011). Some types of privacy and security options that were particularly useful to survivors were those that enabled them to safely and privately use alternate devices (e.g., using private browsing on someone else’s device), effectively control their digital traces (e.g., delete content), and maintain ambiguity and/or plausible deniability in their use of technology (Finn & Atkinson, 2009; Freed et al., 2017; Matthews et al., 2017; Tarzia et al., 2017).

Other common practices that survivors used included limiting or avoiding *sharing personal information online* (e.g., social number security, personal and family pictures, Google account information, credit cards; Dimond et al., 2011; Freed et al., 2017; Matthews et al., 2017). Some opted to shut down some of their online accounts or to delete content and activity histories. Strategies used to achieve control over online presence extended to *physical devices* as well. Survivors threw away their devices (e.g., mobile phones), used alternative devices, changed their SIM card or internet service provider, performed a factory reset on their device, and turned off services like location tracking and Wi-Fi (Freed et al., 2017; Matthews et al., 2017).

### Use of Digital Support Tools and Services

A range of digital support tools and services were made use of by those experiencing IPV, to empower them and increase their safety, engage in screening for IPV with professional agencies, and access online support from those with similar experiences.

Five studies reported on digital interventions which helped to *empower* individuals experiencing IPV and keep them *safe* (Bacchus et al., 2016; Bloom et al., 2014; Choo et al., 2015; Constantino et al., 2007; Finn & Atkinson, 2009). For example, the Online Safety Planning Intervention by Bloom et al. (2014) is a tool designed to provide pregnant abused women with additional strategies on their individualized safety plans (e.g., considerations for escape planning in isolated areas) based upon their self-reported residency. A further example of digital safety planning is offered by Lindsay et al. (2013): The Safety Decision Aid Smartphone Application provides personalized safety plan suggestions based on the user’s responses to questions in the interactive app. For example, if a user indicates in the “My Relationship” section that their partner uses social media to harass them, the personalized safety plan may include detailed information about protecting internet accounts and limiting access to, or closing, these accounts until they feel safe. Additionally, if a user’s “Danger Assessment” score indicates an extreme level of danger, the suggested safety strategies that are offered are worded more urgently to indicate the importance of taking action (Lindsay et al., 2013).

Other approaches involve training individuals on computer safety and other specific technologies, in order to ensure privacy: For example, how to secure a computer against spyware which can monitor computer usage, how to turn off GPS which can be used to track a person’s movements and real-time location, and how to protect baby monitors from being hacked into and thus avoid one’s home being surveilled remotely. This training has been shown to be effective in helping participants to feel safer (Finn & Atkinson, 2009). Another digital tool, an email device called MIVO, was found to be useful as “an email interaction device among women, their child and a nurse to reduce their risk for further interpersonal violence/abuse and to increase disclosure of abuse,” and to provide support and information (Constantino et al., 2007).

Digital tools were also used to *screen for IPV*. For instance, women presenting at hospital emergency departments found that divulging partner abuse via a computer-based screening tool was therapeutic and empowering, and many felt that the computer made it easier to report their experiences compared to face to face interaction (Choo et al., 2015). Similar results were reported by Bacchus et al. (2016) who evaluated the use of the Domestic Violence Enhanced Home Visitation Program (DOVE) program to screen for IPV in pregnant women using computer tablets. DOVE eliminated the complex process for those experiencing IPV of waiting for the right moment in the relationship to ask about or disclose abuse. This was advantageous to women in terms of being able to access help quickly. A further advantage of the computer tablet was its built-in safety mechanism: an icon switched from the DOVE program to a baby video in the case of an unexpected interruption, such as the perpetrator coming home. Only the home visitor could reactivate DOVE with their unique identification number. The greater sense of anonymity and privacy afforded by DOVE in using a computer tablet (compared to face-to-face interviews) meant that women were more likely to answer questions openly around the nature of the abuse that they were experiencing (Bacchus et al., 2016).

Tarzia et al. (2017) report that younger women who experience IPV prefer *online support services* delivered via websites and apps to face-to-face communication for the provision of embarrassing or sensitive information. The anonymity of these online services afforded a more objective and unbiased perspective than they might receive from known friends and family. They also identify important benefits associated with online support services, of convenience, flexibility, low cost, and ability to fill service gaps. However, they also note the need to design such services with the involvement of service users and to attend carefully to factors such as language, tone, anonymity, and links to sources of face-to-face support in service design, in order to encourage uptake (Tarzia et al., 2017).

More broadly, web search tools such as Google were used by survivors to search for information, including general technology information such as learning about new apps, and more specific information—for example, online privacy and safety specific searches (Bloom et al., 2014; Finn & Atkinson, 2009). Survivors also sought out the information provided by IPV support organizations, including high-level summaries of how to think about digital privacy and safety, guides about privacy settings for Facebook, and discussion of security practices such as picking strong passwords (Freed et al., 2017; Lindsay et al., 2013; Tarzia et al., 2017). They found it acceptable to seek advice on IPV via computers/mobile devices, particularly when social supports were unavailable or when information needed to be accessed privately and safely.

### Stalking and Surveillance

Perpetrators secretly extended their abusive behaviors via the internet, through electronic surveillance and stalking/harassment. They also extended preexisting coercive behavior online via sexting coercion (Ross et al., 2016).

*Stalking* was undertaken in a number of ways. Perpetrators monitored their partner’s^3^ social media activity, by constantly checking their profile for updates, waiting for them to come online, looking at the photos their partner had posted, and reading their News feed (Brem et al., 2015; Chaulk & Jones, 2011; Fox & Tokunaga, 2015). They monitored their partner’s connections with others, by visiting the groups that their partner had joined, checking out the events their partner planned to attend and the friends he or she had recently added, and using Facebook to “keep tabs” on their partner and/or their family. Perpetrators also monitored their partner’s location, checking their status on social media to see where they would be (Burke et al., 2011; Finn & Atkinson, 2009; Freed et al., 2017; Matthews et al., 2017; Reed et al., 2016; Truman, 2011; Woodlock, 2017) and by using GPS devices to monitor their real-time location (Freed et al., 2017; Truman, 2011). Chaulk and Jones (2011) found that perpetrators’ online stalking and relational intrusion was frequently facilitated by Facebook. Even when a partner blocked the perpetrator from their Facebook account, the perpetrator may continue their monitoring via the Facebook pages of shared friends, family, or even their children (Brem et al., 2015; Burke et al., 2011; Dimond et al., 2011; Fox & Tokunaga, 2015; Woodlock, 2017).

*Surveillance* and “monitoring”’ are terms that are used interchangeably by researchers in the included studies. In the context of IPV, perpetrators’ *surveillance* of past activities and communications involved checking call histories, email histories, and mobile phone bills (Finn & Atkinson, 2009; Woodlock, 2017). Snooping through a partner’s private communications and messages was achieved by using their passwords to log in to their online accounts without their knowledge (Marcum et al., 2017) or by hacking into their computers and mobile phones (Freed et al., 2017; Reed et al., 2016), and email accounts (Rothman et al., 2009). Using spyware was the most common tactic used by perpetrators in order to monitor their partners. This did not always go undiscovered: Several survivors reported finding spyware on their computer or phone (Burke et al., 2011; Freed et al., 2017; Matthews et al., 2017; Truman, 2011; Woodlock, 2017). Surveillance of physical activity and interactions was undertaken using web cameras, cameras hidden in the home, spyware installed on the partner’s computer, and listening devices/bugs (Burke et al., 2011; Dimond et al., 2011; Truman, 2011; Woodlock, 2017). Monitoring activities could also be less direct: For example, perpetrators could spy by pretending to be the victim/survivor in a chat room or email conversation (Finn & Atkinson, 2009; Woodlock, 2017).

### Effect of Demographic Variables on Identified Strategies

Research Question 3 asked how the strategies identified in Research Question 1 and Research Question 2 are affected by age and gender, and how they varied across non-Western and diaspora populations. Due to the sensitive topic of the review, the majority of the included studies lacked adequate information about demographic characteristics such as gender, age, and geographical region. It was therefore difficult to explore the relationship between strategies used and the demographics and draw general conclusions. Although some of the studies included sufficient demographic characteristics, only half of them included both genders, with women outnumbering men. Only one study included information about the effect of age and geographical region on identified strategies.

Truman (2011) reported that those stalked by intimate partners are significantly younger than those stalked by known others and unknown offenders. Age was significantly and positively associated with higher scores on the seriousness of stalking scale. The same study reported that race/ethnicity (Black and other, non-Hispanic) and stalking type (cyberstalking and stalking with technology) were significant. Both Black and other, non-Hispanic stalking victims had significantly higher odds than White, non-Hispanics of defining the behaviors they experienced as stalking (Truman, 2011). However, given these findings are only from one study, they must be interpreted with caution.

Women were significantly more likely to monitor partners’ behaviors by checking call histories, checking email histories, checking SNSs, using partner’s password to monitor electronic communication, sending excessive emails, and making excessive calls. Conversely, women were significantly more likely to report a partner’s use of technology, such as hidden cameras or GPS, to monitor their behavior (Burke et al., 2011; Truman, 2011). Marcum et al. (2017) indicated that university students who reported participating in cyberstalking via attempted log-ins to their partner’s social media were more likely to be male. According to Reed et al. (2016), there were no gender differences in the number of digital dating abuse behaviors experienced; however, women reported more digital media use overall. Moreover, women were more likely than men to be coerced into sexting. Women reported higher rates and more frequent sexting coercion compared with men, and engaged in more sexting unwillingly. These data suggest that women may be even more likely to “give in” to pressures to sext than to have unwanted but consensual intercourse (Ross et al., 2016). There was a lack of information on other important demographics of interest such as marital status, sexual orientation of the couple, and citizenship status and this warrants future inquiry. Similarly, further research on the influence of age and ethnicity is also warranted.

### Quality Assessment

The majority of the qualitative studies stated the aims of the research clearly (*n* = 8) used appropriate recruitment strategy (*n* = 8) and considered relevant ethical issues (*n* = 7). In addition, they included sufficiently rigorous data analysis (*n* = 8), stated the findings clearly (*n* = 7), and discussed the contribution of the study and the generalizability of research findings (*n* = 8). The majority of the studies (*n* = 7) did not consider the relationship between the researcher and participants adequately. Only five studies collected the data in a way that addressed the research issues. All included studies used appropriate qualitative methodology (Supplemental Material Table C1).

The majority of the cross-sectional studies presented their aims clearly (*n* = 8), included appropriate study design (*n* = 10), defined the target population clearly (*n* = 7), measured appropriately the risk factor and outcome variables (*n* = 10), described the basic data adequately (*n* = 7), and presented the results for all the analyses described in the methods (*n* = 9). In addition, they included well-justified discussions and conclusions (*n* = 9) and discussed the limitations of the studies (*n* = 10). Only half of the studies included a sample frame taken from an appropriate population base so that it closely represented the target/reference population under investigation; measured the risk factor and outcome variables correctly using instruments/measurements that had been trialed, piloted, or published previously; included information about ethical approval. None of the studies included sample size justification section nor measurements to address and categorize nonresponders nor described information about nonresponders. Sample size justification is crucial as sample size profoundly affects the significance of the outcomes of the study. Moreover, nonresponse bias occurs if the nonresponders are substantially different from the rest of the population in the sample. Thus, any information on nonresponders is crucial. Furthermore, only Truman (2011) used an appropriate sampling frame. It is very important that the sampling frame is representative of the target population as results from the study are going to be used to make assumptions about the target population (Supplemental Material Table C2).

The RCT study (Bloom et al., 2014) was judged as having a high risk of bias, while the NRS (Finn & Atkinson, 2009) was judged as weak. The last two sections of the Quality Assessment Tool for Quantitative Studies—the interview integrity and the analysis—were also assigned a quality rating of weak.

## Discussion and Conclusion

### Strategies

This review sought to understand what covert online strategies survivors and perpetrators deploy with respect to IPV. A total of 22 studies (nine qualitative studies, 11 cross-sectional studies, one RCT, and one NRS) were included in the evidence synthesis. The majority of studies were conducted in the United States. Notably, there was a substantial increase in relevant published studies from 2010 onward. Four covert strategies were identified: *presence online*, *granular control* and *use of digital support tools*, *and services* were used by survivors, while *stalking and surveillance* were used by perpetrators of IPV. The strategy of presence online encompassed access to a computer, social connection and support, and restricted presence. It is important to note that while survivors can access support and advice online, they may avoid going online for fear of their perpetrator pursuing them into this space. At a time when interpersonal communications, economic activity and public services are all increasingly conducted online, survivors’ fear of being online disadvantages them and may remove access to social, informational, and practical support. If they did go online, survivors could adopt fine-grained strategies of control over their online presence, by concealing their identities and location, blocking contact from their perpetrators, strengthening privacy settings, restricting the content that they posted, and changing their digital devices in various ways. Survivors’ use of digital support tools and services could help to empower them and increase their safety, engage them in screening for IPV with professional agencies, and provide access to online information and support from professionals and from those with similar experiences. While the internet can thus be seen as a potential “force for good,” it can also be used by perpetrators to secretly extend their abusive behaviors, through digital surveillance, stalking/harassment, and sexting coercion.

### Strengths and Limitations

A strength of this systematic review is the comprehensive search strategy used, which facilitated a more evidence-based approach to literature searching in a field where this is not standard practice. Moreover, the inclusion of study designs other than quantitative studies gave a wide and diverse range of evidence. In the present systematic review, we also included “gray” literature. Another important strength is the use of diverse methodological quality assessment tools, to assess the risk of bias of the included qualitative, quantitative, and mixed-methods studies.

We acknowledge the limitations regarding the number of studies and the methodological quality of studies included. An important limitation of this work is the lack of research on the most current technologies. Most of the studies that passed our screening criteria involved the use of Facebook. However, other social media platforms are increasingly being used (e.g., Instagram, Twitter, WhatsApp, Snapchat), and we do not yet know how they are used either by perpetrators or those experiencing IPV. In addition, studies not in English were excluded from the study, which may bias the findings. As such, the results should be interpreted with some caution.

### Future Work

The review highlighted the need for more well-designed studies that address covert strategies. We need robust research that delivers insights into IPV survivors’ and perpetrators’ online covert behaviors and activities with regard to demographic characteristics, effects on physical and mental health outcomes, and use of a wider range of social media services (e.g., Instagram, WhatsApp). These understandings can help practitioners to gather a more nuanced contemporary picture of survivors’ experiences of IPV and to develop advice for survivors that reflects current digital behaviors. They can also assist law enforcement agencies to be aware of new routes for gathering forensic evidence on abusive behaviors (Nelson, 2019) and inform new legislation on IPV so that it factors in online behaviors.

Finally, we highlight the need for engagement between those who design digital technologies and those with expertise around IPV, to ensure that the design of digital technologies takes account of the risks that can surface for those experiencing IPV. While there is growing interest within the human–computer interaction community in designing technologies to respond to sensitive contexts and events (Chancellor et al., 2019; Herron et al., 2016; Moncur, 2013), partnership with knowledgeable practitioners and those with lived experience is vital in shaping the appropriate design of digital technologies.

## Summary of Practice, Policy, and Research Implications

### Stakeholders: Practitioners (law enforcement, government agencies)

Recommendations: Training and resources for law enforcement to identify covert strategies deployed by individuals engaged in intimate partner violence (IPV), either abusers or victims, as well as enablers and barriers encountered in using these strategies.

Collaboration between police and service sectors to provide support and advice to victims.

Training and resources to provide support and advice to victims.

### Stakeholders: Policy makers

Recommendations: Introduction of specific criminal and civil legislation on online covert behaviors regarding IPV.

### Stakeholders: Researchers

Recommendations: More well-designed studies that address strategies for secret-keeping.

More scientifically assured methods for measuring and analyzing targeted outcomes, in relation to demographic characteristics.

Further research regarding the effects on physical and mental health outcomes due to online covert behaviors and activities.

## Supplemental Material

Supplemental Material, sj-pdf-1-tva-10.1177_1524838020957985 - An Evidence Synthesis of Covert Online Strategies Regarding Intimate Partner Violence Click here for additional data file. Supplemental Material, sj-pdf-1-tva-10.1177_1524838020957985 for An Evidence Synthesis of Covert Online Strategies Regarding Intimate Partner Violence by Aikaterini Grimani, Anna Gavine and Wendy Moncur in Trauma, Violence, & Abuse

## Notes

1. Throughout this article, we apply gender-specific terms *only* where they originate in the papers included in our review, to accurately reflect research content.

2. We excluded studies that exclusively centered on young populations (children and adolescents under 16). However, studies with children and adolescents under 16 that happened to also include adults have been included.

3. In using the term “partner” here, we refer to either a current partner who remains in the abusive relationship or an ex-partner who is no longer in the relationship yet the perpetrator seeks to continue their abusive behavior toward them.
