# Supplementary material for: An Evidence Synthesis of Covert Online Strategies Regarding Intimate Partner Violence
Source: Trauma Violence Abuse. 2020 Sep 15;23(2):581–93. doi: 10.1177/1524838020957985 (PMC8905127; doi:10.1177/1524838020957985)
Supplement: Supplemental Material, sj-pdf-1-tva-10.1177_1524838020957985 - An Evidence Synthesis of Covert Online Strategies Regarding Intimate Partner Violence [file sj-pdf-1-tva-10.1177_1524838020957985.pdf]

Supplemental material

Tables A1-A4. Experiencing or perpetrating Intimate Partner Violence (IPV)

Table A1. Characteristics of the included studies

Study; Country; Type of Methodology	Key themes; Aim	Context and setting	Sampling approach	Participant characteristics	Data collection methods	Data analysis approach	Data collected	Ethical issues
Bacchus et al., 2016; USA; *Qualitative study*	Strategies, Facilitators;<br>• To explore perinatal home visitors' and women's experiences of screening for IPV and receiving DOVE in the form of either mHealth technology (i.e., a computer tablet) or a home visitor-led method.	47 women enrolled to the DOVE trial who had consented to participating in a qualitative interview. Interviews with women took place in their homes if it was safe to do so, or away from the home in the researcher's car. We invited 45 home visitors at the 8 study sites to participate in an interview, which was conducted at their office. The 2 designers of the DOVE computer tablet were interviewed together via Skype.	Purposeful sampling using maximum variation to select women	26 women between 16 and 35 years old; White, African/ African American/ black, Mixed ethnic origin, speaking English or Spanish; 27% Urban, 73% Rural; 4% 7th to 9th grade, 27% 10th to 12th grade, 27% High school graduate/GED, 38% Some college or trade school, 4% College graduate; 69% had experienced IPV in year before current pregnancy while 31% hadn't experienced IPV in year before current pregnancy. Of the 23 home visitors, 9 were from urban sites and 14 were from rural sites. The age range was between 25 and 66 years.	Between November 2013 and August 2014, the first author (LJB) conducted semi-structured interviews with perinatal home visitors and women enrolled in DOVE. Interviews lasted between 1 and 2 hours and used a topic guide that explored a wide range of areas.	Interviews were digitally recorded and transcribed verbatim. NVivo 10 software was used. Thematic analysis was used to identify, analyse, and report on patterns within the data. The initial coding framework in NVivo was guided by the interview schedule themes and was deductive. Deeper exploration and interrogation of the data was inductive, allowing additional themes and their subcategories to emerge.	51 participants (23 home visiting staff, 26 women, and 2 DOVE computer program designers) and 4 nonparticipant observations were conducted	The study was approved by the University of Virginia Institutional Review Board for Social and Behavioural Sciences and the European Union ethics review panel.
Bloom et al., 2014; USA; *RCT*	Strategies, Facilitators;	Recruitment was from community settings (not IPV	Convenience sampling of	46 women: mean age 25.4 years (range = 18–35 years). Family	Women accessed the tool from their own safe computer;	Descriptive statistics	46 women ultimately completing	The University of Missouri Institutional

• To establish feasibility (usability, safety, and acceptability) of the adapted tool for both rural and urban pregnant abused women and practicality of recruitment procedures for future trials.	shelters), targeting women not already accessing formal IPV resources, distributing about 500 recruitment flyers in settings where pregnant women seek services. Ads were posted online, and classified print ads were placed in rural papers.	women (randomly selected)	income for 91.3% was <$2,000 a month from all sources (e.g., employment, gifts, social services), and 47.8% had children under the age of 18 years residing with them. Approximately three of four were White, and over half (56.6%) had at least some college education. 41% of the participants (n = 19) resided in a county designated as nonmetropolitan by the USDA 2003 Rural–Urban Continuum codes	two rural Missouri health departments were partnered with to install computers in private areas that women could use if needed. Potential participants made contact by a toll-free number or e-mail.<br>Women were randomized to intervention or control conditions and provided password-protected access to the tool. All completed self-report measures of IPV exposure, safety behaviours, decisional conflict, and IPV-related health outcomes and received emergency safety plans. Intervention women completed the DA and the priority setting activity and received tailored safety plans. Women were asked to complete 4 online sessions (early pregnancy, late pregnancy, 3 and 6 months postpartum calculated within the tool based upon her due date), with gift cards sent after		baseline sessions online	Review Board reviewed and approved study procedures

					each session via e-mail or letter to the safe address of their choice.			
Bosch and Schumm, 2004; USA; *Cross-sectional study*	Strategies, Facilitators;<br>• How supportive and non-supportive persons helped or hindered their access to resources, and whether access to resources influenced the women's ability to become free from abuse.	42 of the 105 counties in Kansas met initial study criteria. Randomly selected 10 of the 42 Kansas counties; community agencies; local restaurants, laundromats, ice cream shops, convenience stores, grocery stores, donut shops, motels, and beauty shops; ads in local county newspapers	Randomly selected. 1/3 of the participants were recruited with the assistance of the staff of various community agencies who had contact with rural women in abusive partner relationships. Other contacts were made by the interviewer, who used a variety of sources (local restaurants, laundromats, ice cream shops etc.). 3 participants were interviewed as a result of ads placed in local county newspapers	The average age of subjects was 40.5 years (SD = 8.6), with a range of 22 to 63 years. The majority of the subjects were Caucasian (84%), and some were Native Americans (14%) or African-Americans (2%). Most of their former partners were Caucasian (82%), Hispanic (11%), or Native American (7%).<br>*Additional demographics: education, marital status, minor children, Household income, occupation, residency in rural areas, religious affiliation, geographical location and isolation, experience of abuse (p.359-361)*	face-to-face interviews with women	Data was analysed SPSS. Means, standard deviations, and frequencies were computed for items describing geographic or social isolation. A paired samples t-test to compare former and current levels of abuse. Pearson correlation coefficients of the independent and dependent variables. Multiple linear regression procedures, using backwards elimination of variables, to examine the relationship of each of the independent variables to abuse during the relationship and to abuse at the time of the interview.	56 participants (women)	Ethical issues haven't been taken into consideration
Brem et al., 2015; USA; *Cross-sectional study*	Strategies, Barriers;<br>• To examine the display of mate-retention tactics in an online, social-networking environment and how these online	Introductory psychology courses at a small, public, liberal arts university in North Texas; class and campus announcements	141 participants (53 men, 88 women) were recruited from introductory psychology courses at a small, public, liberal arts university in	Participants' ages ranged from 18 to 48 years. The mean age of the men was 20.3 years, the mean age of women was 20.5 years, and 6 participants (3.4%) were above the age of	Data were collected in two waves: 1) a packet of pencil-and-paper questionnaires and 2) online data collection via Survey Monkey.	Descriptive Analyses; exploratory factor analysis with varimax rotation; hierarchical multiple regression analyses	177 participants (112 women; 65 men)	The procedures used for the study were approved by the university's committee for the protection of human subjects

	behaviours are associated with relationship aggression.		North Texas to complete a pencil-and-paper questionnaire. 36 additional participants (12 men, 24 women) were recruited from class and campus announcements where they were given a link to complete an online version of the questionnaire	29 years. The mean relationship length was 18.2 months. The sample consisted of 115 European American (65%), 22 African American (12.4%), 26 Hispanic (14.7%), 7 Asian (4%), 6 Caribbean or West Indian (3.4%), and 1 American Indian (.6%). 172 participants identified as heterosexual (97.2%), 3 participants identified as bisexual (1.7%), and 2 participants identified as gay male or lesbian (1.1%). The mean length of time spent on Facebook on a typical day was 50.8 min.				
Burke et al., 2011; USA; *Cross-sectional study*	Strategies, Barriers;<br>• Do college students use various communication technologies to monitor or control partners in intimate relationships?<br>• Are college students the victim of such monitoring or controlling behaviours?<br>• What are the demographics of	A large southeastern university	Convenience sampling Participants were recruited from a personal health course required for graduation. Researchers used in-class and online announcements to ask these students to complete the anonymous survey.	A sample of 804 participants. Ages ranged from 18 to 23 (M = 19.12) years. Regarding race, 77% of the sample self-identified as white, 14% as black, and 9% as ''other'' (Latino, Asian–American, and American Indian). Of those indicating gender, 500 participants (67.1%) were females and 245 (32.9%) were males, compared to the university's profile of	Responses were collected online using Qualtrics Survey Software. Receipts were generated when completed surveys were submitted. Participants received extra-credit points by presenting these printed receipts to their health instructors.	Content and face validity of the instrument were established; The data were subjected to a Principal Component Analysis (PCA); reliability measures; Cronbach's coefficient alpha was used to determine internal consistency reliability.	804 participants (532 women & 272 men); (a 62% response rate)	The survey, consent form, and research protocols were approved by the university's Institutional Review Board.

	students who are both the initiators and the recipients of this type of behaviour?<br>• What are the validity and reliability of the CPI- Self/ Partner scale?			62% female and 38% male.				
Chaulk and Jones, 2011; Canada; *Cross-sectional study*	Strategies, Barriers;<br>• To determine whether or not online social networks provide an environment in which relational intrusive behaviour can occur and, if so, to examine the form in which this o-ORI occurs.	A large Canadian university	Respondents recruited from the email lists of an undergraduate business faculty at a large Canadian university.	230 respondents that included an even distribution of males and females, the majority of whom were between the ages of 18 and 25.	Each respondent was randomly assigned to one of 6 groups based on two variables (to report either their behaviours as “doers” of the o-ORI tactics or the behaviours of others towards them as “receivers” of the o-ORI tactics.	Frequency analysis	230 participants	Ethical issues haven’t been taken into consideration
Choo et al., 2015; USA; *Qualitative study*	Strategies, Facilitators;<br>• To explore women’s’ attitudes about use of computers for screening and intervening in drug use and partner abuse.	Emergency department	Convenience sampling (of day and evening, weekday and weekend shifts in order to capture the full range of ED visits)	Adult female patients between the ages of 18 and 65 years fluent in English; mean age was 30.6 years (median 28.5 years, range 18 to 50 years). 10 women non-white: 4 black/African American, 2 Hispanic/Latino, one Asian, 1 American Indian/Alaska Native and 2 mixed race. 2 high school but did not graduate; 7 high school or a general educational	Semi-structured interviews: A survey, administered on a tablet-style computer (iPad), contained questions on a broad range of health-related topics. Those who qualified for the study & provided written informed consent were scheduled for an interview date	An integrated set of codes, consisting of all mutually agreed-upon codes, was entered into the NVIVO database with the final version of each transcript	17 participants (women)	The Institutional Review Board (IRB) of the participating hospital approved all study procedures.

				development (GED) certificate; 7 currently in college; 1 a college degree. 8 single/never married, 3 married, 1 separated, 2 divorced, and 3 a member of an unmarried couple. 8 had children.				
Constantino et al., 2007; USA; *Qualitative study*	Strategies, Facilitators; • To test the feasibility of an email device called MIVO for use in interacting with women and children after receiving Protection from Abuse	The Neighbourhood Legal Services Association (NLSA)	Subjects were recruited via announcements placed at NLSA waiting room.	A majority of the six mother and child pairs was White, non-Hispanic (75%, n = 9) and the rest were African Americans. The women were between 30 and 45 years old and the children were from 11 to 13 years of age. Most of the women completed high school, and three women had college degrees	Qualitative phenomenological Design; An interventionist trained both mother and child how to use 'MIVO', an Internet-connected computer (55–70 min orientation included a 5-min practice session).	Thematic Analysis The process of corroboration, or triangulation method was used to ensure consistency and guard against bias; The analytic steps specifically included open coding, axial coding and selective coding followed by summarization	Sample included six mothers who have received a PFA within the past 6 months. (*The sample included six mother and child pairs (n=12) but only adults' responses were taken into consideration*)	Consent forms were obtained; No further information about ethical approval
Dimond et al., 2011; USA; *Qualitative study*	Strategies, Barriers; • To explore how domestic violence survivors have been affected in any way with the addition of new ICTs.	A domestic violence shelter in the southern United States	Convenience sampling; An employee who was the first author's mentor during volunteering spread word that we were conducting research interviews concerning domestic violence and technology.	6 women were African American, 2 were white, 1 was Caribbean, and 1 was from Sub-Saharan Africa; Age between 20s and 50s.	The interviews reflect methods used in feminist participatory action research	Thematic Analysis Authors transcribed the interviews from audio; identified general patterns of experience and subthemes in the patterns	10 women	Consent forms were obtained; No further information about ethical approval
Finn and Atkinson, 2009; USA;	Strategies, Facilitators;	Domestic violence agencies	Participants at domestic violence agencies were	Age ranged from 15 to 74 years, with a median age of 35	Technology education and training was	Descriptive statistics; T-test	729 domestic violence service participants	Ethical issues haven't been

NRS	• To discuss the development, implementation, and evaluation of a model program to provide technology awareness, knowledge, and skills to victims of domestic violence through a training program at domestic violence victim service offices and shelters in Washington State		asked to participate in the training as well as in the evaluation (Non-randomized)	years and a mean of 36.2 years. Most of the respondents were white (66.7%); African American (3.4%), Asian/Asian Pacific American (2.7%), Hispanic (16.7%), Native American/Alaskan Native (5.9%), Multiracial (3%) and "Other" (.6%). 35.1% was single, 30% divorced, 28.5% married, 4.7% "partnered," and 1.7% widowed.<br>75.6% of respondents have children.	provided at 18 domestic violence victim service agencies in Washington State—five, seven, and six agencies respectively over a 3-year period. (pre-test survey and post-test survey)		(women). Pre-tests were filled out by 479 respondents and 387 (80.8%) filled out post-tests	taken into consideration
Fox and Tokunaga, 2015; USA; *Cross-sectional study*	Strategies, Barriers;<br>• To test a model synthesizing attachment, investment model variables, and post-dissolution emotional distress as predictors of interpersonal surveillance of one's ex-partner on Facebook after a breakup	A large Midwestern university and	Convenience sampling (were offered course credit for completing the survey)	Participants aged 18–42 years (M= 20.34 years); White/ European/European-American (n = 342; 79.4%); Black/ African/ African American (n = 21; 4.9%); Asian/Asian-American (n = 25; 5.8%); Latino/a/ Hispanic (n = 10; 2.3%); multiracial (n = 25; 5.8%); or other (n = 5; 1.2%); Heterosexual (n = 415; 96.3%), gay/ lesbian (n = 8; 1.9%), or bisexual (n = 7; 1.6%). One participant declined to report sexual orientation.	Online survey	Confirmatory factor analysis using SAS v9.4	431 participants (150 male and 281 female) Facebook users	Institutional Review Board approval was obtained for the study

Freed et al., 2017; USA; *Qualitative study*	Strategies, Facilitators, Barriers;<br>• To develop a nuanced understanding of the role played by digital technologies in the IPV ecosystem	4 Family Justice Centers	The Deputy Director helped us by making a wide range of professionals aware of the opportunity to participate in our study. To recruit survivors, the Deputy Director placed fliers (in English and Spanish) that described the study in the FJC reception area.	Professionals: 35 Female, 5 Male; Age between 22 & 56 (average 33 y); 16 Case managers/case workers, 10 Social workers, 8 Attorneys/ paralegals, 6 Police officers<br>Survivors: 32 females; Age between 25 & 55 (average 35 y); From Argentina, Dominican Republic, Ecuador, Egypt, El Salvador, Guatemala, Honduras, Jamaica, Mexico, Peru, Russia, UK, USA; 5 did not complete high school, 10 completed high school, 5 attended college, did not graduate, 7 completed college, 5 unreported	Semi-structured interviews (nine focus groups with 32 survivors)	Thematic analysis	72 participants: 40 IPV professionals and nine focus groups with 32 survivors of IPV	Authors received IRB approval for all study procedures and permission from the OCDV before beginning the research
Halligan et al., 2013; USA; *Cross-sectional study*	Strategies, Barriers;<br>• To assess the degree to which individuals involved in abusive relationships would report that technology in the form of text messages, email, and Facebook would impair their ability to terminate the relationship.	A large southeastern university	Convenience sampling	The majority of respondents (81%) were female, white (67% with 19% black, 12% Hispanic) and heterosexual (91%). Over half (64%) were either first or second year undergraduates with the mean age 19.8.	A 42 item Internet questionnaire	Descriptive statistics	259 undergraduates	Study was approved by the Institutional Review Board at a large south-eastern university

Lindsay et al., 2013; USA; *Qualitative study*	Strategies, Facilitators; • To explore a prototype smart phone application (“app”) that is a safety decision aid for female survivors of dating violence.	Craigslist, Facebook, student listservs, campus flyers, and word-of-mouth were used to recruit female college students who identified as survivors of dating violence.	Convenience sampling	Participants from all four research sites were English-speaking female college students, ages 18–25 (M = 21.26), who reported that they experienced dating violence while in college. Participants’ self-identified racial/ ethnic background as 52.6% White, 23.7% Hispanic /Latina, 7.9% African American, 13.2% Multiracial, and 2.6% Other. All participants were currently college students—the majority was undergraduate, including 5.2% freshman, 13.2% sophomores, 21.1% juniors, 28.9% seniors, and 10.1% graduate students.	Focus group discussions or individual interview	Content analysis Transcripts were uploaded into Dedoose (http://www.dedoose.com/) qualitative software to facilitate the qualitative descriptive analysis.	38 participants (women)	Study procedures were consistent across all sites and approved by IRBs at Johns Hopkins University, Arizona State University, University of Missouri-Columbia, and Oregon Health and Science University.
Matthews et al., 2017; USA; *Qualitative study*	Strategies, Barriers; • To provide a framework for organizing survivors' technology practices and challenges into three phases: physical control, escape, and life apart.	Agencies served homeless adults and survivors of IPA (intimate partner abuse)	Agency staff recruited participants through personal contact	*No demographic information due to a privacy precaution*	Semi-structured interviews	Inductive analysis	15 participants (14 female, 1 male)	All participants gave informed consent; the incentives were approved by the agencies and our organization’s internal ethics review
Marcum et al., 2017; USA;	Strategies, Barriers; • To investigate theoretical	A mid-sized university in the Southeast	A sample of 5000 undergraduate and graduate students	The sample was 28 % female. The average	Survey	Descriptive statistics;	890 fully completed surveys of	Consent forms were obtained; No

Cross-sectional study	predictors of cyberstalking in a sample of university students by applying General Theory of Crime and Social Learning Theory.		was randomly chosen by the university's Office of Institutional Research, Assessment, and Planning.	age of the sample was 18 to 20 years old. Eighty 9 % of the sample was white. The average sexual orientation was heterosexual.		correlation analysis; regression analysis	individuals in relationships were returned (17.8 % response rate)	further information about ethical approval
Reed et al., 2016; USA; *Cross-sectional study*	Strategies, Barriers;<br>• To explore whether digital media are a context and tool for dating violence among college students by assessing victimization and perpetration of potentially abusive digital dating behaviours, examining gender differences in these behaviours, and comparing the experience of digital behaviours to other forms of dating violence.	An introductory psychology course at a large university in the Midwestern United States	Convenience sampling	Participants ranged in age from 17-22 (M = 18.66), with the majority (87.9%) being 17, 18, or 19. Although most participants identified their ethnicity as White (72.1%), others identified as Asian (14.8%), Black (6.8%), or Hispanic/Latino (3.8%).	Surveys were administered in paper-and-pencil form to participants in groups of approximately 10 people, seated spaced out around the room, who were given clipboards to use for added privacy. Participants were asked to place their survey in a brown envelope before returning it to the experimenter to further ensure anonymity.	Descriptive statistics; t-test; chi square; Anova	365 undergraduate students (57% female)	Consent forms were obtained; No further information about ethical approval
Ross et al., 2016; USA; *Cross-sectional study*	Strategies, Barriers;<br>• A closer examination of the relation between sexual and sexting coercion, as well as between coercion victimization and sexual behaviour,	A midsized, Midwestern university	Convenience sampling	Most were college freshmen (62%) or sophomores (25%), with an average age of 20.43 years (SD = 4.64). Most were Caucasian (83%), followed by Hispanic (5%), African American (5%), Asian (4%), and 3% who	Online survey	Chi square; correlations; MANCOVA	885 undergraduates (301 men and 584 women)	Consent forms were obtained; No further information about ethical approval

	and determining whether sexting coercion is associated with negative outcomes for victims, above and beyond what is reported by those who experience more traditional forms of IPA exclusively.			identified as biracial, Native American, or "other."				
Rothman et al., 2009; USA; *Cross-sectional study*	Strategies, Barriers; • To estimate the prevalence of e-mail use among residents of battered women's shelters in Massachusetts • To investigate whether, to their knowledge, shelter residents' e-mail accounts had ever been accessed by a nonauthorized intimate partner • To explore whether shelter residents would welcome follow-up contact by researchers or shelters advocates via their e-mail accounts.	11 battered women's shelters in Massachusetts	Convenience sampling	The majority of our sample was in their middle or late 20s (40%), was Black or Hispanic (51%), and did not have a college education (72%)	Survey (16 original questions, 7 demographic and 9 pertaining to e-mail use and comfort with shelter contact via e-mail.	Descriptive statistics	57 participants (women)	Ethical issues haven't been taken into consideration
Tarzia et al., 2017; Australia;	Strategies, Facilitators;	Advertisements on the University of Melbourne	Convenience sampling	Age between 20 and 25 years participated Two of the groups	Semi-structure interviews	Transcripts of the focus groups were	19 participants (women)	Ethics approval for this research was granted by

Qualitative study	• To explore the views of young Australian women on using a website or application to address IPV	online student portal, Twitter, Facebook, Gumtree, Craigslist Australia, and various women's health websites		(n = 4 and n = 5) were open to women aged 16–50, and two were open only to women aged 16–25 (n = 8 and n = 5).	(Four focus groups between April and August 2014)	entered into the software program NVivo 10		The University of Melbourne Human Research Ethics Committee
Truman, 2011; USA; *Cross-sectional study*	Strategies, Facilitators, Barriers; To examine both intimate and nonintimate stalking and the behaviour of cyberstalking	The National Crime Victimization Survey: Stalking Victimization Supplement (United States Department of Justice (USDOJ), Bureau of Justice Statistics (BJS), 2009).	"Rotating panel" design (randomly selected)	The age range of all victims (stalking and harassment victims) was 18 to 90 with a mean of 40.54. Race was coded as white, black, American Indian/Alaska Native, Asian, Hawaiian/Pacific Islander, or multiple races (any combination and up to 4-5 races – this was recoded into one category of multiracial). The majority of all, stalking, and harassment victims were white.	Paper-and-pencil interviewing (both in person and by telephone with the responses entered on a paper instrument) and computer-assisted telephone interviewing	Descriptive statistics; Bivariate analyses; ANOVA; Multiple t-tests; Linear and logistic regression analyses	1,683 victims (67.6% females and 32.4% males)	It has been taken from a pre-existing dataset where consent was obtained
Woodlock, 2017; Australia; *Qualitative study*	Strategies, Barriers; • To determine whether technology-facilitated stalking is an issue for women experiencing domestic violence and whether mobile technologies (such as smartphones)	Authors advertised the victim survey on the DVRCV website, Facebook, Twitter, and gumtree.com.au In addition, they displayed posters at universities and in health centers.	Convenience sampling	The average age of the women was 35 years. 92% (n = 42) identified as Anglo-Australian, 91% (n = 41) as heterosexual, 9% (n = 5) as bisexual, 9% (n = 4) as having a disability, and 37% (n = 17) as a parent with children.	Online surveys (worker survey & victim survey) included closed and open questions, a form of multiple-methods research that captures quantitative and qualitative data	Authors used NVivo to code the answers to the open questions in the two surveys and used thematic analysis to categorize the findings	152 workers in the domestic violence sector & 46 women	Research is guided by internal policies and consultation with external experts to ensure that international ethical standards for working with victims of

	present further opportunities for the perpetration of stalking and domestic violence against women.	domestic violence are followed

Table A2. Description of the covert strategies

Strategy	Type of methodology	Study	Description of the strategy	Evidence of effectiveness
Presence Online	Cross-sectional	(Bosch and Schumm, 2004)	Access to resources such as computer/ internet/ e-mail	Access to resources was associated significantly and negatively with previous abuse, whether correlated with previous abuse or used to predict previous abuse in the regression model. Thus, we can infer that most of the independent variables would have an indirect impact on previous abuse, operating through access to resources. (p. 366)
		(Chaulk and Jones, 2011)	Communication through Facebook	Following: (e.g., Joining the same group(s)/network(s)/ event(s) as you, adding the same applications, checking out the things you have done through your Mini-feed).<br>Intruding uninvited into interactions: (e.g., reading your wall conversations (posts and replies). (p.248) (see Table 5, p.252-253)<br>We find evidence of behaviours identified in the research on stalking and relational intrusion and find that many of them are facilitated by the Facebook application. (p.250)
		(Halligan et al., 2013)	Communication through text messages, e-mail, Facebook	Respondents needing to leave an abusive relationship more frequently identified with those areas that would be perceived as a barrier (e.g. receiving text message that called the respondent names). Hence they were staying in contact with the abusive partner. (p.647)
	Qualitative	(Choo et al., 2015)	Computer Interventions	S4 (age 23): It's like you wanted to know, and you're gonna reflect on my answers, and you're gonna help me out. That's how I see it…It still made me feel like I was somebody, I was a person, I was out there, somebody seen me, like that's—it was yeah. Made me feel good about myself.<br>Three participants described therapeutic or empowering experiences when divulging partner abuse on the computer. (p.4)

		In fact, many participants felt that the computer made it easier to discuss these topics than face-to-face with a person. One participant (S14, age 20) stated that if a doctor asked her about partner abuse, "I tell them that's not what I came here for," but answered the screening questions "because it was on the computer." (p.5)
(Dimond et al., 2011)	Communication through Facebook	Although she does not put any information on Facebook, Janelle tells us that she still visits her sister's page, to see what she is doing and to feel more in touch. Janelle feels that her sister updates her page more frequently for Janelle's benefit.(p.416) In a time of isolation and separation from their social network, sites such as Facebook provide survivors with much needed connection to family and friends. (p.419)
(Freed et al., 2017)	Limited access/ use of computer/ internet	"[Google is] just trying to track me . . . Literally I can go back for months and see where I was at. I had to disable everything. I would not even put information in my phone for a Google account. I'm back to using a paper calendar." (P2, Client) (p.11)
(Lindsay et al., 2013)	Personalized safety plan	Participants were receptive to the "My Safety" portion of the app. The instant nature of the feedback and the color-coded levels that indicate level of danger in an abusive relationship were particularly helpful: "I like how it's broken down like that: extreme danger zone, severe danger zone, increased danger zone, variable danger zone, from red to greenish." Women also liked the scoring: What helped me out was the last, the scoring, how it scores you. It asks you specific information or specific questions about it and then it gives you a score. I think that helped me just to see what the level was. Participants found the "My Plan" section of the app an important resource for safety, stating that creating a plan with resources seemed helpful: "The whole idea of having a plan would be really useful." (p.379) Participants thought that the "My Plan" section was a good way to bring all of the information "together at the end because it's assessing everything and then ends with a plan, which puts all that into perspective." (p.383)
(Matthews et al., 2017)	Limited access/ use of computer/ internet	Due to abusers' physical control of devices, multiple survivors limited or avoided using devices and/or accounts the abuser could access. "I simply stopped using the laptop at home. And the phone. That's why I went to the library to use the computer." An ongoing practice in life apart was to limit or avoid sharing information online. "With my [child], I'll put a picture up, but I just make sure I chop the background, [...] the last picture I posted, [my child] was at [city], we were at the [city place]. You

				can't really tell what [place] it is. [...]. You could see cement and chairs, but you can't really see the background." -P5 (p.2196)
		(Tarzia et al., 2017)	Websites and applications	Websites and apps were perceived as being more objective and unbiased than friends or family (p.206)<br>Maybe have a forum where people post what they're going through, that could give a sense that I'm not going through this alone. There are so many people who are going through the same thing. (FG 1); I feel like young women nowadays always sort of go to the Internet first, just to check out … is this normal? … It's nicer just to know there are other people out there who are asking these questions as well. (FG 2) (p.206)
Granular Control	Qualitative	(Dimond et al., 2011)	Using alias	When Janelle arrived at the shelter, she also got a new prepaid cell phone, but registered it under an alias, rather than using her real name. (p.416)<br>''Yeah so one day I just went and got a new metro and so it is so easy to get a metro, you don't even have to an ID and stuff. . .you can go in and say you are Miss Beyonce Knowles and they don't care.'' (p. 418)<br>For Gina and Janelle, it was not worth the risk to use their real names again, as they feared that their information could show up on other phones or on the Internet. (p.418)
		(Freed et al., 2017)	Limited access/ use of computer/ internet	"[Google is] just trying to track me . . . Literally I can go back for months and see where I was at. I had to disable everything. I would not even put information in my phone for a Google account. I'm back to using a paper calendar." (P2, Client) (p.11)<br>Common software advice included: Limiting or restricting the sharing of pictures, blocking other users on social media and deleting or shutting down online accounts. Common hardware advice included: throwing away the device, changing the SIM card or wireless plan, performing a factory reset on the device, and turning off services like location and WiFi (p.14)
		(Matthews et al., 2017)	Limited access/ use of computer/ internet	Due to abusers' physical control of devices, multiple survivors limited or avoided using devices and/or accounts the abuser could access. "I simply stopped using the laptop at home. And the phone. That's why I went to the library to use the computer."<br>An ongoing practice in life apart was to limit or avoid sharing information online. "With my [child], I'll put a picture up, but I just make sure I chop the background, [...] the last picture I posted, [my child] was at [city], we were at the [city place]. You can't really tell what [place] it is. [...]. You could see cement and chairs, but you can't really see the background." -P5 (p.2196)

				Some types of privacy and security options that were particularly useful to survivors were those that enabled them to safely and privately use alternate devices (e.g., using private browsing on someone else's device), effectively control their digital traces (e.g., delete content), and maintain ambiguity and/or plausible deniability in their use of technology (p.2197)
		(Tarzia et al., 2017)	Protect privacy	These included password protection for the app or website; disguising the app or website as something unrelated to IPV; automatic log out of the app or website if a woman does not use her phone within a set period of time; and providing information to users about how to clear their cache or browser history. (p.210)
	NRS	(Finn and Atkinson, 2009)	Anonymous accounts and keeping secret the location	Sending email without giving away your location; Creating anonymous email account that does not give out your private information (Table 4; p.57)
	Cross-sectional	(Chaulk and Jones, 2011)	Facebook privacy settings	Users of social networking sites are well-advised to be selective in their criteria for adding 'friends' and be wary of individuals that begin to exhibit higher forms of relational intrusion behaviours. For example, Facebook allows one to restrict the majority of their profile to friends only. Currently, Facebook does allow users to block some individuals and to limit profile viewing to others.<br>"Ever since I had Facebook my privacy settings have been on the highest setting. Unless I have accepted the person as my friend, they cannot see my profile. I only accept friend requests from people I know to a degree higher than simply knowing their name. Unfortunately, people are not as careful with their privacy as I am." (p.251-252)
		(Truman, 2011)	Block mechanisms	Change e-mail address; installed caller ID or call blocking; changed or installed new locks or security system (Table 8; p.59)<br>Victims of intimate partner stalking installed caller ID or call blocking ($\chi2(1)=7.39$; $p<.01$), and changed or installed new locks or security system ($\chi2(1)=21.25$; $p<.001$). (p.79)
Use of digital support tools and services	RCT	(Bloom et al., 2014)	Online Safety Planning Intervention	The tool is designed to provide women with additional rural-specific strategies on their individualized safety plans (e.g., considerations for escape planning in isolated areas) based upon their input.<br>Three of four participants (73.9%) logged in and completed the baseline session within a week of enrolment, with 45.7% completing within 24 hours. The average time to completion was 10.3 days (SD = 16.3 days, range = 0–68 days), with rural

			women taking an average of 2.2 days longer than urban women (11.6 vs. 9.4 days, respectively).<br>The average DA score was 16.1, which indicates severe danger in the abusive relationship, with rural women two points higher on average (17.3; SD = 9.6, range = 1–33) than urban women (15.3; SD = 9.5, range = 1–34). RAs reported that rural women were also typically able to identify fewer safe emergency contacts (friends or family members who could safely take a message for her) than urban women. (p.248)
NRS	(Finn and Atkinson, 2009)	Technology Safety Project	Many respondents reported having increased awareness about computer safety and better understanding of how to stay safe. In addition, they reported gaining new or additional knowledge about specific technologies, especially spyware, GPS, cell phones, and baby monitors. Several mentioned that they would change some specific computer and technology behaviour as a result of the presentation. (p.57)<br>“I won’t put my real name when signing up for services online” and “I’m going to take personal information off accounts and pass information on to residents in the shelter.” (p.57)<br>The presentation was positive for many and made them feel safer and more protected. This knowledge was also “scary” for many respondents. Often there was a mix of fear and appreciation of the knowledge. For example, one respondent said, “I’m more scared now, but it’s good to know this stuff.” Another stated, “I will not use the computer as much.” (p.57)<br>Approximately 80% of respondents found information in all areas to be useful, with the exception of information about Instant Messenger, on which more than half (59.1%) found the information useful. (Table 5; p.56-57)
Qualitative	(Bacchus et al., 2016)	DOVE technology	“DOVE really helped a lot…Some women could tell you right off the bat “look he beat me.” But some women could be just like me and it takes time. I think if they do it and the home visitor comes in and they’re graceful and supportive, I think it will help [women] a lot. I feel like it helped me a lot and to trust people again.” [Joanne, client, 21 years, rural, IPV+] (p.7)<br>The DOVE technology eliminated this complex process of waiting for the right moment in the relationship to ask about or disclose abuse, which was advantageous to women in terms of being able to access help quickly. (p.9)<br>An advantage of the computer tablet was its built-in safety mechanism, an icon that switched from the DOVE program to a baby video in the case of an unexpected interruption. This safety feature was greatly appreciated because only the home visitor

		could reactivate DOVE with his or her unique identification number. (p.12)
(Choo et al., 2015)	Computer Interventions	S4 (age 23): It's like you wanted to know, and you're gonna reflect on my answers, and you're gonna help me out. That's how I see it…It still made me feel like I was somebody, I was a person, I was out there, somebody seen me, like that's—it was yeah. Made me feel good about myself.<br>Three participants described therapeutic or empowering experiences when divulging partner abuse on the computer. (p.4)<br>In fact, many participants felt that the computer made it easier to discuss these topics than face-to-face with a person. One participant (S14, age 20) stated that if a doctor asked her about partner abuse, "I tell them that's not what I came here for," but answered the screening questions "because it was on the computer." (p.5)
(Constantino et al., 2007)	e-mail device "MIVO" intervention	Results indicated that email was a feasible and acceptable way to provide support and information to abused women after obtaining a PFA order.<br>All six mother and child pairs emailed the nurse interventionist at least once a week; two mothers sent email three times during both the third and fourth weeks (p.296)<br>"What do I do if he comes to pick up his belongings without police escort?"; "If police refuses to escort him, can he still pick up his belongings?"; "If he calls me at work, should I call the police?" (p.296)
(Freed et al., 2017)	Protect privacy	Changing passwords and password recovery questions, changing privacy settings (e.g., for Facebook), inspecting the device for unwanted apps (often by taking it to an official company store like the Apple store) (p.14)
(Lindsay et al., 2013)	Personalized safety plan	Participants were receptive to the "My Safety" portion of the app. The instant nature of the feedback and the color-coded levels that indicate level of danger in an abusive relationship were particularly helpful: "I like how it's broken down like that: extreme danger zone, severe danger zone, increased danger zone, variable danger zone, from red to greenish." Women also liked the scoring: What helped me out was the last, the scoring, how it scores you. It asks you specific information or specific questions about it and then it gives you a score. I think that helped me just to see what the level was. Participants found the "My Plan" section of the app an important resource for safety, stating that creating a plan with resources seemed helpful: "The whole idea of having a plan would be really useful." (p.379)

				Participants thought that the “My Plan” section was a good way to bring all of the information “together at the end because it’s assessing everything and then ends with a plan, which puts all that into perspective.” (p.383)
		(Tarzia et al., 2017)	Websites and applications	Websites and apps were perceived as being more objective and unbiased than friends or family (p.206)<br>Maybe have a forum where people post what they’re going through, that could give a sense that I’m not going through this alone. There are so many people who are going through the same thing. (FG 1); I feel like young women nowadays always sort of go to the Internet first, just to check out … is this normal? … It’s nicer just to know there are other people out there who are asking these questions as well. (FG 2) (p.206)
Stalking and Surveillance	Cross-sectional	(Brem et al., 2015)	Facebook surveillance (mate-retention tactic)	90.1% of the sample reported looking at a partner’s Facebook page; 39.5% checked a partner’s statuses to see where he or she would be; 33.1% snooped through a partner’s private messages and/or chat. (p.2837)<br>Items on the Jealousy and Surveillance subscale significantly predicted the occurrence of both psychological aggression (β = .30, p = .003) and physical assault (β = .26, p = .02) over and above the effects of offline mate-retention tactics. (p.2843)
		(Burke et al., 2011)	Online monitoring behaviours: Checking call histories; Checking email histories; Checking mobile phone bills; Monitoring partner’s Facebook site; Using GPS device to monitor partner; Using web cams to monitor partner; Using hidden cams to monitor partner; Using spy ware to monitor partner; Using partner’s passwords to monitor him/her	See Tables 2; 3; 4; 5 (p.1165-1166)
		(Chaulk and Jones)	Facebook surveillance	Monitoring: (e.g., constantly checking your profile for updates, waiting for you to come online, visiting the groups you’ve joined, checking out the events you'll be attending and the friends you've recently added, using Facebook to "keep tabs" on you and/or your family, looking at the photos you have posted, reading your Mini-feed).<br>Covertly obtaining information: (e.g., using Facebook profile to obtain information about you, using the profiles of family/friends/co-workers to obtain information about you). (p.248) (see Table 5, p.252-253)<br>For example, more than half responded that they had used Facebook to make contact with ex-intimates. Further, almost a third responded that they used Facebook to “keep tabs” on the person and to obtain information about the person’s activities by reading their wall and profile. These types of behaviours

		represent a form of monitoring and surveillance which is consistent with lower forms of obsessive relational intrusion. (p.250)
(Fox and Tokunaga, 2015)	Interpersonal electronic surveillance through Facebook	Individuals who monitored their ex-partners online immediately following the dissolution of their relationship were more likely to monitor their ex-partners online currently (b = 0.60, SE = 0.03, p < 0.001). (Table 1; p.494)<br>The present results suggest that individuals most traumatized by a breakup are most likely to monitor their ex-partners online (p.495)
(Marcum et al., 2017)	Login to other's social networking account without their knowledge	The average for attempted log-in to other's social networking account without their knowledge is 1.14. In the correlations, low self-control has a link with attempted to login (r = 0.25), but Hirschi's (2004) version of self-control does not have a link with attempted login. Further, peer association does have a link with attempted login (r = 0.01). The regression analysis for low self-control as a correlate of attempted to login. For attempted to login, low self-control has a link (Beta = 0.01). (p.380)
(Reed et al., 2016)	Monitoring behaviours	Results show that some behaviours, especially items such as "Looked at my/my partner's private information on a computer or cell phone without permission," "Monitored my/my dating partner's whereabouts," "Monitored who I/partner talk(s) to and who I/they am/are friends with," are especially common in this sample. (Table 1; p.1564)
(Ross et al., 2016)	Sexting coercion	Correlations between sexual aggression victimization (as measured by the SVAWS Sexual Aggression subscale), sexual coercion victimization, and sexting coercion victimization (both measured via the SCIRS) ranged from .38 to .54 (p < .001 for all) for both men and women. (p.11)
(Rothman et al., 2009)	Access to e-mail	Of the respondents who reported having an e-mail account (n = 27), 77% reported that they did not give permission for anyone else to use their account. The majority of respondents (96%) reported that they checked their e-mail daily (46%) or weekly (38%), as opposed to monthly (4%). Respondents were most likely to report that they typically checked their e-mail from a public place<br>(e.g., the library) (38%) but also reported checking their e-mail while at home (19%), at school (19%), at the homes of friends or relatives (19%), or at work (15%). In total, 81% of respondents reported that they checked e-mail from a place other than their own home (p.740)
(Truman, 2011)	Electronic monitoring	Video or digital cameras; computer spyware; listening devices/ bugs; GPS (Table 5; p. 49)

Qualitative	(Dimond et al., 2011)	Limited access/ use of computer/ internet	Janelle says she used to have a Facebook and MySpace, but now she says, ''My life on the Internet is over.'' Janelle tells me that she used to go on Facebook every day and was very active in posting updates and communicating with her friends and family, but is no longer active on the site due to harassment and threats from her husband and his family (p.416)<br>But she is hesitant to put any information on the Internet and this can be problematic, especially when trying to look for permanent housing, search for jobs, or submit job applications that typically require a social security number (SSN). Janelle says she is particularly afraid to enter her SSN, as she believes her husband may find that she is in a different state.(p.416)<br>Because Tia perceived her ex to have much more technical prowess than her, she said she does not really go online anymore. She also said she is afraid to use credit cards or anything that might track her. (p.418)
	(Freed et al., 2017)	Monitoring behaviours	"Using technology is the fastest and easiest way, I think, for many of our clients' abusers to gain access, because it's so easy — when you've been cohabitating with somebody for so long — to gain access to bank accounts, social security numbers, all of these things via shared devices, phones, and things like that. Many of our clients have to sort of untangle that, as well as figure out which accounts have been compromised." (P31, Case manager)<br>Another common strategy was for the abuser to go through the client's phone when they were not looking, such as while they were asleep or taking a shower. (p.8)<br>"A lot of clients tell me that he keeps getting their password to their Facebook. I tell them it's because he knows all your information. It's easy to get into your password if you know the security questions to answer." (P27, NYPD) (p.9)<br>"He gave my child an iPad and was using that to find out what's going on at home. He would use Facetime, where he gets to see where my child is and maybe what's going on in the background. It's like having him at home again, even though he's not actually there." (P24, Client) (p.9)
	(Matthews et al., 2017)	Monitoring behaviours (Device/account controlled & monitored; Spyware installed; Account hijacked – Impersonated; Account hijacked - Locked out; Account monitored - Remote or unknown means)	Abusers used this physical proximity to monitor survivors' devices and accounts and, in a few cases, install spyware on survivors' devices. (p.2193)<br>Three participants reported that their abusers installed spyware on their devices to monitor their activity. "Then I took the phone to [a store] and they said this phone… somebody put something

			in the phone and this person can see everything you—where you call, who you talk to, all the logs." -P2 (p.2195)<br>In life apart, abusers relied on digital attacks, such as account hijacking and online harassment. "Whenever she hacked my [social media] account she messaged everybody in my family that I didn't want her to contact, pretending to be me. [...] And she would try to get them to trust her [when] they definitely shouldn't. [...] Messaging people in my family trying to get more information about me." -P13 (p.2195)
	(Woodlock, 2017)	Monitoring behaviours	Used text messages, phone, and so on to call her names, harass her, or "put her down"; Used mobile technology to check her location; Impersonated her in emails, text messages, and/or social media; Tracked her via GPS (e.g., using applications such as Find My Friends); Checked her text messages without her permission (Table 1; p. 591)<br>My client's ex-partner has tracked her down after following her Facebook use. He assaulted her, stole her phone, and accessed her Facebook [account]. He has changed her passwords, and she is now not able to access her own account. He is contacting all her friends and supports, pretending to be her. This has resulted in her becoming very isolated. He has allegedly sent sexual messages to male friends in her account, resulting in the client feeling ashamed and powerless. The client has reported the incident, but police have not been able to find the respondent. (p.596)
NRS	(Finn and Atkinson, 2009)	Monitoring behaviours	25.1% of women had their browser history monitored; 23.6% had been repeatedly threatened, insulted or harassed by email; 18% had someone monitor their emails, and 17% had someone use their PIN or password to gain access to a private email account; 11.5% had someone pretended to be them in an email using their personal account. 9.4% had someone pretended to be them in a chat room using their personal screen name; 9.4% had someone used their Social Security number or financial information to buy things online without their knowledge (p.56)

Table A3. Description of the technology which enables concealed and supportive behaviours related to IPV

Facilitator	Type of methodology	Study	Description of the facilitator	Evidence of facilitator
Digital devices	Cross-sectional	(Bosch and Schumm, 2004)	Computer with access to the internet/ e-mail	Access to resources is facilitated by a variety of types of support and appears to reduce abuse during the partner relationship and, indirectly, when partners are no longer living together. (p.367)
	RCT	(Bloom et al., 2014)	Computer	The most common self-reported location of the safe computer used for both rural and urban women was at home. A higher percentage of rural women (63.2%) reported using a home computer compared with their urban counterparts (48.4%), and a lower percentage of rural women used a computer at a friend's or family member's house (25.3% vs. 34.3%, respectively) (p.248)
	NRS	(Finn and Atkinson, 2009)	Computer	The vast majority of the 479 respondents (92.9%) has access to and uses a computer. Of those, 58.7% use a computer at home, 43.6% at the library, 39.2% at work, 14.4% at a community center, and 38% use a computer at another location (such as a friend's house, domestic violence organization or coffee shop). (p.55)<br>55% of respondents view the computer as an important way to research safety. (Table 2; p.55)
	Qualitative	(Bacchus et al., 2016)	Computer tablet (facilitates trust establishment; offers a greater sense of anonymity and privacy)	"If I did not trust her I would not have done this…if that were the case then I would like the tablet. Then I could have answered the questions and I don't have to worry if she had seen them." [Kimberley, client, 20 years, rural, IPV+]<br>The computer tablet appeared to offer women a greater sense of anonymity and privacy, thereby encouraging more openness in answering the abuse questions.<br>"There are just some things you feel ashamed saying, no matter how trustworthy that person…And with a computer there's no emotion…and you can just say whatever you need to say and you won't feel like you're being judged…it was like a security blanket." [Lisa, client, 20 years, rural, IPV+]<br>"A lot of people don't like to talk and express themselves so [the computer] brings it more out of a person even if they're afraid." [Jennifer, client, 30 years, urban, IPV+]<br>"Maybe us asking those questions could be the first time it's ever been brought up. So if they feel safe enough to do it on the tablet, feeling like it's a little anonymous, it starts to break down those walls and maybe next time they'll want to talk about it." [Coleen, home visitor, 27 years, rural] (p.8)

		(Choo et al., 2015)	Computer; mobile	Computers/mobile devices were described as acceptable and accessible for drug use and partner abuse advice, particularly when social supports are unavailable or when information must be accessed privately and safely. (p.6)<br>Over half (eleven of seventeen) of the participants said they frequently used a computer or mobile device, describing a wide variety of reasons for use, including entertainment, social networking, and general information. The social connections, in particular, seemed important to the women; one stay at home mom (S15, age 27), described her mobile device as her only connection to the outside world. Ten of seventeen participants stated they used the computer/mobile device for health information for themselves or friends or family. Importantly, a few women defined distinctions between mobile device and computer use, with computers associated with productivity, school and work, and cell-phones related to social connections and immediate, practical functions such as finding places and getting needed facts or information. (p.8)
		(Constantino et al., 2007)	MIVO (e-mail device)	The mothers and their children utilized the MIVO without difficulty. Feedback from the mothers and children was quite positive. (p.296)
		(Lindsay et al., 2013)	Mobile device/ smartphone	College-age women would respond positively to a technology-based safety decision aid, such as this, that could be accessed from a smartphone or other mobile device. (p.385)
		(Tarzia et al., 2017)	Smartphone	Most of the young women highlighted the benefits that the Internet and smartphones could offer in terms of providing ready access to support when experiencing IPV. "If you have your phone with you, that means it opens up a lot …. You don't have to go to a friend, you don't have to go to a safe place or a specific place to access information, it can be 24/7, anywhere you are, which is great. (FG 3) (p.210)
Digital tools and services	Cross-sectional	(Truman, 2011)	Digital tools and services	Change e-mail address; installed caller ID or call blocking; changed or installed new locks or security system (Table 8; p.59)
	NRS	(Finn and Atkinson, 2009)	Phone and computer-related services	The vast majority of respondents are comfortable using phone and computer-related services. Very few are comfortable with GPS, TTY/TDD services, or PDAs. (Table 1; p. 55)
	Qualitative	(Freed et al., 2017)	Google search	A common strategy for learning about technology was simply searching the web for information during or between client meetings. This could be both for general technology information, such as learning about new apps, or for digital privacy/safety specific problems, such as how to prevent location tracking. (p.12)

		"I get most of my safety tip sheets from the NNEDV, the Safety Net Project. So if they have something that's helpful, great, I can print it out and give it to a client. But if they don't, then we're kind of trying to figure it out, and so I'm like Googling stuff." (P14, Staff Attorney)<br>These organizations provide a number of useful documents, including high-level summaries of how to think about digital privacy and safety, guides about privacy settings for Facebook, and discussion of security practices such as picking strong passwords. (p.12-13)
(Lindsay et al., 2013)	Mobile application	Participants generally agreed that using a mobile phone application, such as this, was a useful, innovative, and effective way to get information to college students about dating violence and safety in relationships.<br>"It helps you organize your mind because when you're in the situation, you don't really know how to feel … there's so much going on, you don't really know how to categorize things. When [the app does] it for you, it just helps you put yourself in order, and have more control on your life.<br>When something's happening to you like that, you feel like you're out of control and you can't—you don't know where to go. You don't know what to do. You're just so confused, so I think it helps. (p.378)
(Matthews et al., 2017)	Software	"[A software product] let me know when someone's trying to hack into my account. Then I used the [2-factor authentication] method and I change the password. So that is so cool for me. It's a couple times. I think the last time was my ex. You know he thought he could just check my email and see what I'm doing." - P11 (p.2196)
(Tarzia et al., 2017)	Web applications	The young women felt that seeking help anonymously via the Internet or an app would be easier in many ways than face-to-face. (p.205)

Table A4. Description of the technology which deters concealed and supported behaviours related to IPV

**Barrier**	**Type of methodology**	**Study**	**Description of the barrier**	**Evidence of barrier**
Social networking sites	Cross-sectional	(Brem et al., 2015)	Facebook	90.1% of the sample reported looking at a partner's Facebook page; 39.5% checked a partner's statuses to see where he or she would be; 33.1% snooped through a partner's private messages and/or chat. (p.2837)<br>Items on the Jealousy and Surveillance subscale significantly predicted the occurrence of both psychological aggression (β = .30, p = .003) and physical assault (β = .26, p = .02) over and above the effects of offline mate-retention tactics. (p.2843)
		(Burke et al., 2011)	Facebook	Monitoring partner's Facebook site (Tables 2; 3; 4; 5) (p.1165-1166)
		(Chaulk and Jones, 2011)	Facebook	We find evidence of behaviours identified in the research on stalking and relational intrusion and find that many of them are facilitated by the Facebook application. (see Table 1; 3) (p.250)
		(Fox and Tokunaga, 2015)	Facebook (or similar social networking sites)	SNSs and other mediated interpersonal channels will continue to play integral roles in the escalation, maintenance, and dissolution of romantic relationships. (p.496)
		(Marcum et al., 2017)	Social media	The average for attempted log-in to social media is 1.14. In the sample, 96 % of the sample used social media or social network sites. (p.380)
	Qualitative	(Dimond et al., 2011)	Facebook (or similar social networking sites)	Sites such as Facebook were also used as an extension of abuse after leaving for three of the women. As we learned from Janelle in the opening story, her abuser used Facebook to harass, threaten, and to try to get information. Thus, technologies can be helpful, but sites such as Facebook do not easily provide the kind of privacy that domestic violence survivors require. (p.417)
		(Freed et al., 2017)	Social media	"Especially in intimate partner cases, there is a lot of overlap in people's social circles. A lot of family overlap, especially if there are children in common. So now with social media you get a lot of influence being put on people. For example, now the abuser's family is posting on Facebook, 'Why'd you get my brother locked up? Why'd you get my son locked up?' " (P28, Attorney)<br>"I try to make sure that when I post it is only seen by my friends, but I realized that there is also a setting where it could be seen by friends of friends. I want to turn that off but I do not know how." (P9, Client) (p.9)<br>In addition to enabling abusers to search for information on how to abuse, the Internet also enables abusers to find information that can help them locate a victim. Personal and organizational

				websites, blogs, social media platforms, and more, often provide names, photos, contact information, and other details. (p.10)
		(Woodlock, 2017)	Facebook	Workers and victims wrote of perpetrators using social media, specifically Facebook, to relentlessly monitor and abuse women. Even when a woman blocks her partner or ex-partner from her Facebook account, he may continue to monitor her through the Facebook pages of shared friends, family, or even their children (p.593)<br>Workers identified Facebook as a platform that perpetrators use to proxy stalk women. (p.594)
Digital devices	Cross-sectional	(Bacchus et al., 2016)	Computer tablet (might affect interpersonal communication; technological issues; Concerns about confidentiality)	"You know if in fact relationship building is so crucial…you know my only concern was the client goes off, they complete the forms, they do all the work on the tablet themselves. They hand the tablet back. Would the community worker truly sit and still have communication with that client or would they have let the tablet do all the work for them…would there be a loss in that relationship?" [Program designer 02] (p.9)<br>"You need to use your tablet often for the tablet to keep connectivity with the Google Play Store and sometimes these tablets sit in a drawer and they miss updates because they're turned off. They lose the token that Google gives the tablet to keep it authenticated…If you don't have that token you will not access the market, you cannot get your update. So skipping updates is really bad when you're dealing with this kind of research software…I would have given them a bit more network knowledge. We didn't teach them about that…I mean they're nurses and they're not supposed to know those things. [Program designer 01]<br>"Well I kinda had this thought in my head…what if it's not going to the people they said it's going to and then he does find me and then I'm screwed…If I were to tell somebody [in person], I think it would go directly to that person or the people that need to know about it. But with the tablet, technology's kinda finicky sometimes and it has glitches and you don't really know where it's going." [Lisa, client, 20 years, rural, IPV+]<br>"Everything you put on the computer everyone can see it. It's probably better letting the home visitor do it because Miss Laura [home visitor] said that if somebody tries to ask her about me, she can't tell them." [Amy, client, 16 years, rural, IPV+] (p.12)
		(Burke et al., 2011)	Monitoring devices	Using GPS device to monitor partner; Using web cams to monitor partner; Using hidden cams to monitor partner (Tables 2; 3; 4; 5) (p.1165-1166)

	(Halligan et al., 2013)	Phone	There was no significant difference (p-values > 0.05) between abused and abuse prone groups in terms of technology access and knowledge of phone features. Almost 58% of total respondents agreed or strongly agreed with the notion that they can't be separated from their cell phones. However, respondents needing to leave an abusive relationship more frequently identified with those areas that would be perceived as a barrier (e.g. receiving text message that called the respondent names). Hence they were staying in contact with the abusive partner. And, those who were "abuse prone" were more likely to stay in abusive relationships even though parents and friends disapproved. (p.646-647) Technology does seem to be a barrier for extricating oneself from an abusive relationship. Third, abuse prone individuals seem just as vulnerable to being trapped by technology. (p.647)
	(Reed et al., 2016)	Mobile phone	"My dating partner(s) monitored who I talk to and who I am friends with using the Internet or a cell phone" "I monitored who my dating partner(s) talk to and who he/she is friends with using the Internet or a cell phone" (p.1561)
	(Truman, 2011)	Video or digital cameras; GPS device; listening devices	Video or digital cameras; GPS; listening devices/ bugs (Table 5; p. 49)
Qualitative	(Choo et al., 2015)	Computer	Many did not own a computer/mobile device and could only use one at public places or when they could borrow one. Others possessed outdated equipment with limited capabilities or were unable to pay for an Internet connection. Although none of the women explicitly described partners monitoring or limiting their use of devices as part of abusive/controlling behaviours, one participant (S4, age 23) related being unable to afford to replace her phone after it was broken in a violent argument: "I don't have a phone… when I had my phone it broke because of the, like, arguing and stuff like that, start slamming stuff, throwing stuff, things like that." (p.9)
	(Dimond et al., 2011)	GPS device; Computer; mobile phone	For example, Heather's abuser sent her a text stating that he knew where she was because her phone has GPS on it. Tia told me that her ex-boyfriend had been stalking her and she did not know how he found out where she was, but attributed his computer skills to his ability to stalk her. ''I mean he is very computer savvy. He knows how to make a computer. Literally like from scratch.'' Whether or not the phones had GPS or the abusers were able to track them, participants were not willing to risk their safety by keeping their phones. (p.417)
	(Freed et al., 2017)	Phone	"Using technology is the fastest and easiest way, I think, for many of our clients' abusers to gain access, because it's so easy

				— when you've been cohabitating with somebody for so long — to gain access to bank accounts, social security numbers, all of these things via shared devices, phones, and things like that. Many of our clients have to sort of untangle that, as well as figure out which accounts have been compromised." (P31, Case manager)Another common strategy was for the abuser to go through the client's phone when they were not looking, such as while they were asleep or taking a shower. (8)<br>"[…] Like, my phone is very hot, the battery disappears constantly. They think, 'Well, I just need a new phone'. No. Probably someone has spyware on your phone, but you don't know that." (P28, Attorney). (p.10)
		(Woodlock, 2017)	Monitoring devices (i.e. GPS)	An additional key finding was the use of GPS mobile technology to engender this sense of omnipresence. Perpetrators usually achieve this by downloading mobile applications ("apps") to women's phones or hiding a GPS device in their vehicles. A participant in the worker survey illustrated the numerous ways in which perpetrators use GPS (p.593)
Digital tools and services	Cross-sectional	(Burke et al., 2011)	Monitoring tools and services	Checking call histories; Checking email histories; Using spy ware to monitor partner; Using partner's passwords to monitor him/her (Tables 2; 3; 4; 5) (p.1165-1166)
		(Rothman et al., 2009)	e-mail monitoring	Of the 27 respondents who reported having an e-mail account, 1 (4%) reported that she knew that her e-mail account had been broken into by a dating partner, and<br>4 additional respondents were "not sure" if this had ever occurred (15%) (p.741)
		(Truman, 2011)	Spyware	Computer spyware (Table 5; p. 49)
	Qualitative	(Dimond et al., 2011)	Blocking	Blocking unwanted calls and text messages is hard, costs money, depends on the carrier and phone, and is sometimes impossible. In some cases as well, blocking was impossible due to government issued phones. (p.419)
		(Freed et al., 2017)	Monitoring tools (i.e. anti-theft software, activate or deactivate services)	Many clients reported sharing a cellular family plan, with their abuser as the account manager for the plan. In this situation, the abuser is in fact the legal owner of the client's account (and any children's accounts) and can track the devices using anti-theft software, activate or deactivate services, and view billing information containing details of any calls, texts, or charges made to the account. (p.9)<br>One prevalent category of apps that came up a lot in our data were tracking apps, ranging from child-monitoring apps for parents to anti-theft apps like Find my iPhone, all of which are commonly used by abusers to monitor clients. With simple Internet searches yielding a vast amount of information on how

		to abuse—and providing access to tools that facilitate abuse. (p.10)
(Matthews et al., 2017)	Spyware	For example, after P2 found spyware on her phone and laptop, she said: “I simply stopped using the laptop at home. And the phone. That's why I went to the library to use the computer.” Her abuser had installed spyware on it, and she wasn’t convinced that it was clean after she reset it (p.2196)
(Woodlock, 2017)	Spyware	“I suspect he may have installed software onto my iPhone enabling him to have access to my phone calls, text messages, Facebook, e-mails, etc. He sometimes says things or behaves in ways that suggests he knows something via a suspicious means. (p.593)

Table B. Search strategies

Databases	Search terms
Medline (OVID)<br>&<br>HMIC	1. exp Domestic Violence/<br>2. (domestic abuse or domestic violence).mp. [mp=title, abstract, original title, name of substance word, subject heading word, keyword heading word, protocol supplementary concept word, rare disease supplementary concept word, unique identifier, synonyms]<br>3. (intimate partner abuse or intimate partner violence).mp. [mp=title, abstract, original title, name of substance word, subject heading word, keyword heading word, protocol supplementary concept word, rare disease supplementary concept word, unique identifier, synonyms]<br>4. Stalking/<br>5. exp Spouse Abuse/<br>6. exp Intimate Partner Violence/<br>7. coercive control.mp. [mp=title, abstract, original title, name of substance word, subject heading word, keyword heading word, protocol supplementary concept word, rare disease supplementary concept word, unique identifier, synonyms]<br>8. Battered Women/

9. (abus$ adj3 wom#n).mp. [mp=title, abstract, original title, name of substance word, subject heading word, keyword heading word, protocol supplementary concept word, rare disease supplementary concept word, unique identifier, synonyms]
10. (abus$ adj3 spous$).mp. [mp=title, abstract, original title, name of substance word, subject heading word, keyword heading word, protocol supplementary concept word, rare disease supplementary concept word, unique identifier, synonyms]
11. (abus$ adj3 partner$).mp. [mp=title, abstract, original title, name of substance word, subject heading word, keyword heading word, protocol supplementary concept word, rare disease supplementary concept word, unique identifier, synonyms]
12. (partner$ adj3 violen$).mp. [mp=title, abstract, original title, name of substance word, subject heading word, keyword heading word, protocol supplementary concept word, rare disease supplementary concept word, unique identifier, synonyms]
13. (spous$ adj3 violen$).mp. [mp=title, abstract, original title, name of substance word, subject heading word, keyword heading word, protocol supplementary concept word, rare disease supplementary concept word, unique identifier, synonyms]
14. (domestic adj3 (abus$ or violen$)).mp. [mp=title, abstract, original title, name of substance word, subject heading word, keyword heading word, protocol supplementary concept word, rare disease supplementary concept word, unique identifier, synonyms]
15. (relationship$ adj3 violen$).mp. [mp=title, abstract, original title, name of substance word, subject heading word, keyword heading word, protocol supplementary concept word, rare disease supplementary concept word, unique identifier, synonyms]
16. (intimate adj3 violen$).mp. [mp=title, abstract, original title, name of substance word, subject heading word, keyword heading word, protocol supplementary concept word, rare disease supplementary concept word, unique identifier, synonyms]
17. (gender adj3 violence).mp. [mp=title, abstract, original title, name of substance word, subject heading word, keyword heading word, protocol supplementary concept word, rare disease supplementary concept word, unique identifier, synonyms]
18. 1 or 2 or 3 or 4 or 5 or 6 or 7 or 8 or 9 or 10 or 11 or 12 or 13 or 14 or 15 or 16 or 17
19. INTERNET/
20. (online adj3 surveillance).mp. [mp=title, abstract, original title, name of substance word, subject heading word, keyword heading word, protocol supplementary concept word, rare disease supplementary concept word, unique identifier, synonyms]
21. (internet adj3 surveillance).mp. [mp=title, abstract, original title, name of substance word, subject heading word, keyword heading word, protocol supplementary concept word, rare disease supplementary concept word, unique identifier, synonyms]

22. (online adj3 harass*).mp. [mp=title, abstract, original title, name of substance word, subject heading word, keyword heading word, protocol supplementary concept word, rare disease supplementary concept word, unique identifier, synonyms]
23. (internet adj3 harass*).mp. [mp=title, abstract, original title, name of substance word, subject heading word, keyword heading word, protocol supplementary concept word, rare disease supplementary concept word, unique identifier, synonyms]
24. (online adj3 abus*).mp. [mp=title, abstract, original title, name of substance word, subject heading word, keyword heading word, protocol supplementary concept word, rare disease supplementary concept word, unique identifier, synonyms]
25. (internet adj3 abus*).mp. [mp=title, abstract, original title, name of substance word, subject heading word, keyword heading word, protocol supplementary concept word, rare disease supplementary concept word, unique identifier, synonyms]
26. (cyberabuse or cyber-abuse or cyber abuse).mp. [mp=title, abstract, original title, name of substance word, subject heading word, keyword heading word, protocol supplementary concept word, rare disease supplementary concept word, unique identifier, synonyms]
27. (cyberstalk* or cyber-stalk or cyber stalk*).mp. [mp=title, abstract, original title, name of substance word, subject heading word, keyword heading word, protocol supplementary concept word, rare disease supplementary concept word, unique identifier, synonyms]
28. ((facebook or social media) adj3 stalk*).mp. [mp=title, abstract, original title, name of substance word, subject heading word, keyword heading word, protocol supplementary concept word, rare disease supplementary concept word, unique identifier, synonyms]
29. (online adj3 victim*).mp. [mp=title, abstract, original title, name of substance word, subject heading word, keyword heading word, protocol supplementary concept word, rare disease supplementary concept word, unique identifier, synonyms]
30. (internet adj3 victim*).mp. [mp=title, abstract, original title, name of substance word, subject heading word, keyword heading word, protocol supplementary concept word, rare disease supplementary concept word, unique identifier, synonyms]
31. (online adj3 stalk*).mp. [mp=title, abstract, original title, name of substance word, subject heading word, keyword heading word, protocol supplementary concept word, rare disease supplementary concept word, unique identifier, synonyms]
32. (internet adj3 stalk*).mp. [mp=title, abstract, original title, name of substance word, subject heading word, keyword heading word, protocol supplementary concept word, rare disease supplementary concept word, unique identifier, synonyms]

33. digital technolog*.mp. [mp=title, abstract, original title, name of substance word, subject heading word, keyword heading word, protocol supplementary concept word, rare disease supplementary concept word, unique identifier, synonyms]
34. (internet adj3 safe*).mp. [mp=title, abstract, original title, name of substance word, subject heading word, keyword heading word, protocol supplementary concept word, rare disease supplementary concept word, unique identifier, synonyms]
35. (online adj3 safe*).mp. [mp=title, abstract, original title, name of substance word, subject heading word, keyword heading word, protocol supplementary concept word, rare disease supplementary concept word, unique identifier, synonyms]
36. technology safety project.mp.
37. computerized decision support.mp.
38. exp Social Media/
39. "Cell Phone Use"/
40. (digital adj3 abus*).mp. [mp=title, abstract, original title, name of substance word, subject heading word, keyword heading word, protocol supplementary concept word, rare disease supplementary concept word, unique identifier, synonyms]
41. social media.mp. [mp=title, abstract, original title, name of substance word, subject heading word, keyword heading word, protocol supplementary concept word, rare disease supplementary concept word, unique identifier, synonyms]
42. (identity adj3 conceal*).mp. [mp=title, abstract, original title, name of substance word, subject heading word, keyword heading word, protocol supplementary concept word, rare disease supplementary concept word, unique identifier, synonyms]
43. (identity adj3 hid*).mp. [mp=title, abstract, original title, name of substance word, subject heading word, keyword heading word, protocol supplementary concept word, rare disease supplementary concept word, unique identifier, synonyms]
44. (cyber-relationship or cyber relationship).mp. [mp=title, abstract, original title, name of substance word, subject heading word, keyword heading word, protocol supplementary concept word, rare disease supplementary concept word, unique identifier, synonyms]
45. (monitor adj3 online).mp. [mp=title, abstract, original title, name of substance word, subject heading word, keyword heading word, protocol supplementary concept word, rare disease supplementary concept word, unique identifier, synonyms]
46. (monitor adj3 internet).mp. [mp=title, abstract, original title, name of substance word, subject heading word, keyword heading word, protocol supplementary concept word, rare disease supplementary concept word, unique identifier, synonyms]

47. (online adj3 privacy).mp. [mp=title, abstract, original title, name of substance word, subject heading word, keyword heading word, protocol supplementary concept word, rare disease supplementary concept word, unique identifier, synonyms]
48. (internet adj3 privacy).mp. [mp=title, abstract, original title, name of substance word, subject heading word, keyword heading word, protocol supplementary concept word, rare disease supplementary concept word, unique identifier, synonyms]
49. human computer interaction.mp.
50. technology-facilitat*.mp. [mp=title, abstract, original title, name of substance word, subject heading word, keyword heading word, protocol supplementary concept word, rare disease supplementary concept word, unique identifier, synonyms]
51. (profile adj3 conceal*).mp. [mp=title, abstract, original title, name of substance word, subject heading word, keyword heading word, protocol supplementary concept word, rare disease supplementary concept word, unique identifier, synonyms]
52. (profile adj3 hid*).mp. [mp=title, abstract, original title, name of substance word, subject heading word, keyword heading word, protocol supplementary concept word, rare disease supplementary concept word, unique identifier, synonyms]
53. social network*.mp. [mp=title, abstract, original title, name of substance word, subject heading word, keyword heading word, protocol supplementary concept word, rare disease supplementary concept word, unique identifier, synonyms]
54. (internet adj3 communicat*).mp. [mp=title, abstract, original title, name of substance word, subject heading word, keyword heading word, protocol supplementary concept word, rare disease supplementary concept word, unique identifier, synonyms]
55. (online adj3 communicat*).mp. [mp=title, abstract, original title, name of substance word, subject heading word, keyword heading word, protocol supplementary concept word, rare disease supplementary concept word, unique identifier, synonyms]
56. (digital adj3 communicat*).mp. [mp=title, abstract, original title, name of substance word, subject heading word, keyword heading word, protocol supplementary concept word, rare disease supplementary concept word, unique identifier, synonyms]
57. pseudonymous.mp. [mp=title, abstract, original title, name of substance word, subject heading word, keyword heading word, protocol supplementary concept word, rare disease supplementary concept word, unique identifier, synonyms]
58. anonymous identit*.mp. [mp=title, abstract, original title, name of substance word, subject heading word, keyword heading word, protocol supplementary concept word, rare disease supplementary concept word, unique identifier, synonyms]

	59. (digital device or phone or laptop or computer or tablet).mp. [mp=title, abstract, original title, name of substance word, subject heading word, keyword heading word, protocol supplementary concept word, rare disease supplementary concept word, unique identifier, synonyms]<br>60. (password manager or encrypt*).mp. [mp=title, abstract, original title, name of substance word, subject heading word, keyword heading word, protocol supplementary concept word, rare disease supplementary concept word, unique identifier, synonyms]<br>61. 19 or 20 or 21 or 22 or 23 or 24 or 25 or 26 or 27 or 28 or 29 or 30 or 31 or 32 or 33 or 34 or 35 or 36 or 37 or 38 or 39 or 40 or 41 or 42 or 43 or 44 or 45 or 46 or 47 or 48 or 49 or 50 or 51 or 52 or 53 or 54 or 55 or 56 or 57 or 58 or 59 or 60<br>62. 18 and 61
ASSIA	((domestic abus*) OR (domestic violen*) OR (intimate partner abus*) OR (intimate partner violen*) OR (coercive control) OR (spous* abus*) OR (spous* violen*) OR MAINSUBJECT.EXACT("Domestic violence")) AND ab(facebook OR (social media) OR (digital technolog*) OR (digital device) OR phone OR laptop OR computer OR tablet OR (password manager) OR encrypt OR pseudonymous OR (anonymous identit*) OR (cyberabuse OR cyber-abuse OR cyber abuse) OR (cyberstalk* OR cyber-stalk OR cyber stalk*) OR (computerized decision support) OR (technolog* safety)) OR ab(internet NEAR/3 (surveillance OR harass* OR abus* OR victim OR stalk* OR safe* OR monitor OR privacy OR conceal* OR communicat*)) OR ab(online NEAR/3 (surveillance OR harass* OR abus* OR victim OR stalk* OR safe* OR monitor OR privacy OR conceal* OR communicat*))
PsycInfo	S65 S17 AND S63<br>S64 S17 AND S63<br>S63 S18 OR S19 OR S20 OR S21 OR S22 OR S23 OR S24 OR S25 OR S26 OR S27 OR S28 OR S29 OR S30 OR S31 OR S32 OR S33 OR S34 OR S35 OR S36 OR S37 OR S38 OR S39 OR S40 OR S41 OR S42 OR S43 OR S44 OR S45 OR

S46 OR S47 OR S48 OR S49 OR S50 OR S51 OR S52 OR S53 OR S54 OR S55 OR S56 OR S57 OR S58 OR S59 OR S60 OR S61 OR S62

S62	password manager or encrypt*
S61	anonymous identit*
S60	digital device or phone or laptop or computer or tablet
S59	anonymous identit
S58	DE "Anonymity"
S57	pseudonymous
S56	digital N3 communicat*
S55	online N3 communicat*
S54	internet N3 communicat*
S53	social network*
S52	profile N3 hid*
S51	profile N3 conceal*
S50	technology-facilitat*
S49	DE "Human Computer Interaction"
S48	internet N3 privacy
S47	online N3 privacy
S46	monitor N3 internet

S45	monitor N3 online
S44	cyber-relationship or cyber relationship
S43	identity N3 hid*
S42	identity N3 conceal*
S41	computerized decision support
S40	DE "Decision Support Systems"
S39	technology safety project
S38	online N3 safe*
S37	internet N3 safe*
S36	digital technolog*
S35	internet N3 stalk*
S34	online N3 stalk*
S33	internet N3 victim*
S32	online N3 victim*
S31	((facebook or social media) N3 stalk*)
S30	cyberstalk* or cyber-stalk or cyber stalk*
S29	cyberabuse or cyber-abuse or cyber abuse
S28	internet N3 abus*
S27	online N3 abus*

S26 internet N3 harass*

S25 online N3 harass*

S24 internet N3 surveillance

S23 online N3 surveillance

S22 DE "Social Media"

S21 DE "Online Community"

S20 DE "Online Social Networks"

S19 DE "Internet Usage"

S18 DE "Internet"

S17 S1 OR S2 OR S3 OR S4 OR S5 OR S6 OR S7 OR S8 OR S9 OR S10 OR S11 OR S12 OR S13 OR S14 OR S15 OR S16

S16 gender N3 violence

S15 intimate N3 violence

S14 relationship N3 (abus* or violence)

S13 domestic N3 (abus* or violence)

S12 spous* N3 violence

S11 violence N3 partner

S10 abus* N3 partner*

S9 abus* N3 spous*

	S8 DE "Battered Females"
	S7 coercive control
	S6 DE "Partner Abuse"
	S5 DE "Stalking"
	S4 intimate partner abuse or intimate partner violence
	S3 domestic abuse or domestic violence
	S2 DE "Intimate Partner Violence"
	S1 DE "Domestic Violence"
ACM Digital Library	Search 1: (+domestic +abuse)
	Search 2: (+domestic +violence)
	Search 3: (+intimate partner +violence)
	Search 4: (+intimate partner +abuse)
	Search 5: (+spouse +abuse)
	Search 5: (+spouse +violence)
	Search 6: (+stalk +partner)
	Search 7: (+stalk +spouse)
	Search 8: cyberstalk
	Search 9: (+harass +partner)
	Search 10: (+harass +spouse)

Social Science Citation Index (via Web of Science)	TS = (((domestic abus*) OR (domestic violen*) OR (intimate partner abus*) OR (intimate partner violen*) OR (coercive control) OR (spous* abus*) OR (spous* violen*) OR MAINSUBJECT.EXACT("Domestic violence")) AND ab(facebook OR (social media) OR (digital technolog*) OR (digital device) OR phone OR laptop OR computer OR tablet OR (password manager) OR encrypt OR pseudonymous OR (anonymous identit*) OR (cyberabuse OR cyber-abuse OR cyber abuse) OR (cyberstalk* OR cyber-stalk OR cyber stalk*) OR (computerized decision support) OR (technolog* safety)) OR ab(internet NEAR/3 (surveillance OR harass* OR abus* OR victim OR stalk* OR safe* OR monitor OR privacy OR conceal* OR communicat*)) OR ab(online NEAR/3 (surveillance OR harass* OR abus* OR victim OR stalk* OR safe* OR monitor OR privacy OR conceal* OR communicat*))) AND TOPIC: ((facebook) OR (social media) OR (digital technolog*) OR (digital device) OR phone OR laptop OR computer OR tablet OR (password manager) OR encrypt OR pseudonymous OR (anonymous identit*) OR (cyberabuse OR cyber-abuse OR cyber abuse) OR (cyberstalk* OR cyber-stalk OR cyber stalk*) OR (computerized decision support) OR (technolog* safety)) OR TOPIC: ((internet NEAR/3 (surveillance OR harass* OR abus* OR victim OR stalk* OR safe* OR privacy OR conceal*))) OR TOPIC: ((online NEAR/3 (surveillance OR harass* OR abus* OR victim OR stalk* OR safe* OR privacy OR conceal*)))

Tables C1-C2. Quality Assessment

Table C1. CASP Tool			
	**No of studies scoring YES**	**No of studies scoring NO**	**No of studies scoring UNCLEAR**
1. Was there a clear statement of the aims of the research?	9 (Bacchus et al., 2016, Choo et al., 2015, Clarke et al., 2013, Constantino et al., 2007, Dimond et al., 2011, Lindsay et al., 2013, Matthews et al., 2017, Tarzia et al., 2017, Woodlock, 2017)	1 (Freed et al., 2017)	-
2. Is a qualitative methodology appropriate?	10 (Bacchus et al., 2016, Choo et al., 2015, Clarke et al., 2013, Constantino et al., 2007, Dimond et al., 2011, Freed et al., 2017, Lindsay et al., 2013, Matthews et al., 2017, Tarzia et al., 2017, Woodlock, 2017)	-	-
3. Was the research design appropriate to address the aims of the research?	4 (Bacchus et al., 2016, Clarke et al., 2013, Constantino et al., 2007, Dimond et al., 2011)	-	6 (Choo et al., 2015, Freed et al., 2017, Lindsay et al., 2013, Matthews et al., 2017, Tarzia et al., 2017, Woodlock, 2017)
4. Was the recruitment strategy appropriate to the aims of the research?	9 (Bacchus et al., 2016, Choo et al., 2015, Clarke et al., 2013, Constantino et al., 2007, Dimond et al., 2011, Freed et al., 2017, Matthews et al., 2017, Tarzia et al., 2017, Woodlock, 2017)	1 (Lindsay et al., 2013)	-
5. Was the data collected in a way that addressed the research issue?	6 (Bacchus et al., 2016, Choo et al., 2015, Clarke et al., 2013, Dimond et al., 2011, Freed et al., 2017, Matthews et al., 2017)	1 (Tarzia et al., 2017)	3 (Constantino et al., 2007, Lindsay et al., 2013, Woodlock, 2017)
6. Has the relationship between researcher and participants been adequately considered?	2 (Clarke et al., 2013, Dimond et al., 2011)	7 (Choo et al., 2015, Constantino et al., 2007, Freed et al., 2017, Lindsay et al., 2013, Matthews et al., 2017, Tarzia et al., 2017, Woodlock, 2017)	1 (Bacchus et al., 2016)
7. Have ethical issues been taken into consideration?	7 (Bacchus et al., 2016, Choo et al., 2015, Freed et al., 2017, Lindsay et al., 2013, Matthews et al., 2017, Tarzia et al., 2017, Woodlock, 2017)	-	3 (Clarke et al., 2013, Constantino et al., 2007, Dimond et al., 2011)
8. Was the data analysis sufficiently rigorous?	9 (Bacchus et al., 2016, Choo et al., 2015, Clarke et al., 2013, Dimond et al., 2011, Freed et al., 2017, Lindsay et al., 2013, Matthews et al., 2017, Tarzia et al., 2017, Woodlock, 2017)	-	1 (Constantino et al., 2007)
9. Is there a clear statement of findings?	8 (Bacchus et al., 2016, Choo et al., 2015, Clarke et al., 2013, Freed et al., 2017, Lindsay et al., 2013, Matthews et al., 2017, Tarzia et al., 2017, Woodlock, 2017)	1 (Constantino et al., 2007)	1 (Dimond et al., 2011)
10. How valuable is the research?	9 (Bacchus et al., 2016, Choo et al., 2015, Clarke et al., 2013, Dimond et al., 2011, Freed et al., 2017, Lindsay et al., 2013, Matthews et al., 2017, Tarzia et al., 2017, Woodlock, 2017)	1 (Constantino et al., 2007)	-

Table C2. AXIS Tool						
	No of studies scoring YES		No of studies scoring NO		No of studies scoring UNCLEAR	
Introduction						
1. Were the aims/objectives of the study clear?	8	(Bosch and Schumm, 2004, Burke et al., 2011, Halligan et al., 2013, Marcum et al., 2017, Reed et al., 2016, Ross et al., 2016, Rothman et al., 2009, Truman, 2011)	2	(Chaulk and Jones, 2011, Fox and Tokunaga, 2015)	1	(Brem et al., 2015)
Methods						
2. Was the study design appropriate for the stated aim(s)?	10	(Bosch and Schumm, 2004, Brem et al., 2015, Burke et al., 2011, Chaulk and Jones, 2011, Fox and Tokunaga, 2015, Halligan et al., 2013, Marcum et al., 2017, Reed et al., 2016, Ross et al., 2016, Rothman et al., 2009)	1	(Truman, 2011)	-	
3. Was the sample size justified?	1	(Truman, 2011)	10	(Bosch and Schumm, 2004, Brem et al., 2015, Burke et al., 2011, Chaulk and Jones, 2011, Fox and Tokunaga, 2015, Halligan et al., 2013, Marcum et al., 2017, Reed et al., 2016, Ross et al., 2016, Rothman et al., 2009)	-	
4. Was the target/reference population clearly defined? (Is it clear who the research was about?	7	(Bosch and Schumm, 2004, Brem et al., 2015, Burke et al., 2011, Marcum et al., 2017, Reed et al., 2016, Rothman et al., 2009, Truman, 2011)	2	(Fox and Tokunaga, 2015, Halligan et al., 2013)	2	(Chaulk and Jones, 2011, Ross et al., 2016)
5. Was the sample frame taken from an appropriate population base so that it closely represented the target/reference population under investigation?	5	(Bosch and Schumm, 2004, Burke et al., 2011, Marcum et al., 2017, Rothman et al., 2009, Truman, 2011)	6	(Brem et al., 2015, Chaulk and Jones, 2011, Fox and Tokunaga, 2015, Halligan et al., 2013, Reed et al., 2016, Ross et al., 2016)	-	
6. Was the selection process likely to select subjects/participants that were representative of the target/reference population under investigation?	1	(Truman, 2011)	6	(Brem et al., 2015, Chaulk and Jones, 2011, Fox and Tokunaga, 2015, Halligan et al., 2013, Reed et al., 2016, Ross et al., 2016)	4	(Bosch and Schumm, 2004, Burke et al., 2011, Marcum et al., 2017, Rothman et al., 2009)
7. Were measures undertaken to address and categorise non-responders?	-		11	(Bosch and Schumm, 2004, Brem et al., 2015, Burke et al., 2011, Chaulk and Jones, 2011, Fox and Tokunaga, 2015, Halligan et al., 2013, Marcum et al., 2017, Reed et al., 2016, Ross et al., 2016, Rothman et al., 2009, Truman, 2011)	-	
8. Were the risk factor and outcome variables measured appropriate to the aims of the study?	10	(Bosch and Schumm, 2004, Brem et al., 2015, Burke et al., 2011, Fox and Tokunaga, 2015, Halligan et al., 2013, Marcum et al., 2017, Reed et al., 2016,	-		1	(Chaulk and Jones, 2011)

		Ross et al., 2016, Rothman et al., 2009, Truman, 2011)				
9. Were the risk factor and outcome variables measured correctly using instruments/measurements that had been trialled, piloted or published previously?	6	(Brem et al., 2015, Burke et al., 2011, Fox and Tokunaga, 2015, Reed et al., 2016, Ross et al., 2016, Truman, 2011)	5	(Bosch and Schumm, 2004, Chaulk and Jones, 2011, Halligan et al., 2013, Marcum et al., 2017, Rothman et al., 2009)	-	
10. Is it clear what was used to determined statistical significance and/or precision estimates? (e.g. p-values, confidence intervals)	5	(Bosch and Schumm, 2004, Chaulk and Jones, 2011, Fox and Tokunaga, 2015, Halligan et al., 2013, Rothman et al., 2009)	1	(Truman, 2011)	5	(Brem et al., 2015, Burke et al., 2011, Marcum et al., 2017, Reed et al., 2016, Ross et al., 2016)
11. Were the methods (including statistical methods) sufficiently described to enable them to be repeated?	2	(Bosch and Schumm, 2004, Rothman et al., 2009)	7	(Burke et al., 2011, Chaulk and Jones, 2011, Fox and Tokunaga, 2015, Halligan et al., 2013, Marcum et al., 2017, Reed et al., 2016, Ross et al., 2016)	2	(Brem et al., 2015, Truman, 2011)
Results						
12. Were the basic data adequately described?	7	(Bosch and Schumm, 2004, Brem et al., 2015, Chaulk and Jones, 2011, Fox and Tokunaga, 2015, Reed et al., 2016, Rothman et al., 2009, Truman, 2011)	4	(Burke et al., 2011, Halligan et al., 2013, Marcum et al., 2017, Ross et al., 2016)	-	
13. Does the response rate raise concerns about non-response bias?	2	(Chaulk and Jones, 2011, Marcum et al., 2017)	1	(Truman, 2011)	8	(Bosch and Schumm, 2004, Brem et al., 2015, Burke et al., 2011, Fox and Tokunaga, 2015, Halligan et al., 2013, Reed et al., 2016, Ross et al., 2016, Rothman et al., 2009)
14. If appropriate, was information about non-responders described?	-		11	(Bosch and Schumm, 2004, Brem et al., 2015, Burke et al., 2011, Chaulk and Jones, 2011, Fox and Tokunaga, 2015, Halligan et al., 2013, Marcum et al., 2017, Reed et al., 2016, Ross et al., 2016, Rothman et al., 2009, Truman, 2011)	-	
15. Were the results internally consistent?	2	(Burke et al., 2011, Truman, 2011)	3	(Reed et al., 2016, Ross et al., 2016)	6	(Bosch and Schumm, 2004, Brem et al., 2015, Fox and Tokunaga, 2015, Halligan et al., 2013, Marcum et al., 2017, Rothman et al., 2009)
16. Were the results presented for all the analyses described in the methods?	9	(Bosch and Schumm, 2004, Brem et al., 2015, Burke et al., 2011, Fox and Tokunaga, 2015, Marcum et al., 2017, Reed et al., 2016, Ross et al., 2016, Rothman et al., 2009, Truman, 2011)	-		2	(Chaulk and Jones, 2011, Halligan et al., 2013)
Discussion						

17. Were the authors' discussions and conclusions justified by the results?	9	(Brem et al., 2015, Burke et al., 2011, Chaulk and Jones, 2011, Fox and Tokunaga, 2015, Marcum et al., 2017, Reed et al., 2016, Ross et al., 2016, Rothman et al., 2009, Truman, 2011)	2	(Bosch and Schumm, 2004, Halligan et al., 2013)	-	
18. Were the limitations of the study discussed?	10	(Brem et al., 2015, Burke et al., 2011, Chaulk and Jones, 2011, Fox and Tokunaga, 2015, Halligan et al., 2013, Marcum et al., 2017, Reed et al., 2016, Ross et al., 2016, Rothman et al., 2009, Truman, 2011)	1	(Bosch and Schumm, 2004)	-	
Other						
19. Were there any funding sources or conflicts of interest that may affect the authors' interpretation of the results?	-		5	(Brem et al., 2015, Burke et al., 2011, Fox and Tokunaga, 2015, Reed et al., 2016, Ross et al., 2016)	6	(Bosch and Schumm, 2004, Chaulk and Jones, 2011, Halligan et al., 2013, Marcum et al., 2017, Rothman et al., 2009, Truman, 2011)
20. Was ethical approval or consent of participants attained?	5	(Brem et al., 2015, Burke et al., 2011, Fox and Tokunaga, 2015, Halligan et al., 2013, Truman, 2011)	-		6	(Bosch and Schumm, 2004, Chaulk and Jones, 2011, Marcum et al., 2017, Reed et al., 2016, Ross et al., 2016, Rothman et al., 2009)
